# Supplementary material for: Towards a dynamic model to estimate evolving risk of major bleeding after percutaneous coronary intervention
Source: PLOS Digit Health. 2025 Jun 25;4(6):e0000906. doi: 10.1371/journal.pdig.0000906 (PMC12193038; doi:10.1371/journal.pdig.0000906)
Supplement: S1 Table — The highlighted rows are those variables that were removed in the multicollinearity analysis. (DOCX) [file pdig.0000906.s002.docx]

**S1 Table**. Variables included at each stage and their definition as coded in the NCDR data dictionary. The highlighted rows are those variables that were removed in the multicollinearity analysis.

| **Model Stage** | **Definition** |
| --- | --- |
| **Stage 1** |  |
| AA_BetaBlockers | Anti Anginal Type, if given: Beta Blockers |
| AA_CaChannel | Anti Anginal Type, if given: CA Channel |
| AA_LongActingNitrates | Anti Anginal Type, if given: Long Acting Nitrates |
| AA_OtherAgent | Anti Anginal Type, if given: Other Agent |
| AA_Ranolazine | Anti Anginal Type, if given: Ranolazine |
| AdmtSource_1 | Admit Source: ED |
| AdmtSource_2 | Admit Source: Transfer |
| AdmtSource_3 | Admit Source: Other |
| Age | Age |
| AGEGT70 | Age if Greater than 70 |
| AGELE70 | Age if Less than or Equal to 70 |
| AnginalClass | Anginal Classification |
| AntiAnginalMed | Anti-Anginal Meds within 2 Weeks |
| BMI | Body Mass Index |
| BMILE30 | Body Mass Index if Less than or equal to 30. |
| CADPresentation | CAD Presentation |
| CardioLVSD | Cardiomyopathy or LV Systolic Dysfunction |
| CARSHOCK | Cardiogenic Shock on Presentation |
| ChronicLungDisease | Chronic Lung Disease |
| CKD | Chronic Kidney Disease Composite Variable: 1 = Patients with GFR >= 60, 2 Patients with 45 <= GFR < 60, 3 Patients with 30 <= GFR < 45, 4 Patients with Current Dialysis OR GFR < 30 |
| CKD1 | Binary variable if CKD == 1 |
| CKD2 | Binary variable if CKD == 2 |
| CKD3 | Binary variable if CKD == 3 |
| CurrentDialysis | Currently on Dialysis |
| DCARSHOCK | Coded Variable for Prior Cardiac Shock and PCI Cardiac Shock: 1 if Prior Cardio Shock AND PCI Cardio Shock, 2 if Prior Cardio Shock, 3 if no PriorCardioShock AND PCI Cardio Shock, 4 if No Prior CardioShock and No PCICardioShock |
| Diabetes | Has Diabetes |
| DiabetesControl_Diet | If Has Diabetes, control: Diet |
| DiabetesControl_Insulin | If Has Diabetes, control: Insulin |
| DiabetesControl_None | If Has Diabetes, control: No current control |
| DiabetesControl_Oral | If Has Diabetes, control: Oral |
| DiabetesControl_OtherOrMissing | If Has Diabetes, control: Other or Missing |
| Dyslipidemia | Has Dyslipidemia |
| FamilyHxCAD | Family History of CAD |
| FEMALE | Binary variable for sex. |
| GFR | Glomular Filtration Rate: Calculated from Pre Procedure Creatinine, Age, RACEMULT, and GENDMULT |
| HDEF | Pre-PCI LVEF %, if greater than 60, round to 60, then divide all values by 5 |
| Height | Height |
| HispOrig | Patient Race |
| Hypertension | Patient has hypertension |
| InsIHS | Insurance HIS |
| InsMedicaid | Patient Insurance: Medicaid |
| InsMedicare | Patient Insurance: Medicare |
| InsMilitary | Insurance: Military |
| InsNone | Patient Insurance: None |
| InsNonUS | Insurance: Non US-bsed Insurance |
| InsPrivate | Patient Insurance: Private |
| InsState | Insurance: State Provided |
| LYTICS | Binary variable: 1 if CAD Presentation is STEMI and Thrombolytics Given (ThromTherapy==1) |
| NEWDIAB | 0: if patient does not have diabetes, 1 if patient has diabetes and diabetes control is Diet, Oral, Insulin, 2 if patient has diabetes and control is "other" |
| NEWDIAB1 | If NEWDIAB is 1 |
| NEWDIAB2 | if NEWDIAB is 2 |
| NYHA123 | NYHA Classification in past two weeks if past two weeks HF indicated as 1, 2, or 3 |
| NYHA4 | NYHA is 4 |
| OnsetTimeEst | CAD Presentation Symptom Onset Time is Estimated |
| OnsetTimeNA | CAD Presentation Symptom Onset Time is not available |
| PCIStatus | PCI Status |
| PREHGBGT13 | Pre-procedure Hemoglobin is greater than 13 |
| PREHGBLE13 | Pre-procedure Hemoglobin is less than or equal to 13 |
| PrePCILVEF | Pre-PCI LVEF% |
| PrePCILVEFNA | Pre-PCI LVEF% is not available |
| PreProcCKMB | CK-MB Pre Procedure |
| PreProcCKMBNM | Pre Procedure CK-MB, if Calculated, is normal or not? |
| PreProcCreat | Pre Procedure Creatinine |
| PreProcHgb | Pre Procedure Hemoglobin |
| PreProcTnl | Troponin I pre procedure |
| PreProcTnT | Troponin T pre-procedure |
| Prior2weekNYHA | NYHA Classification in prior 2 weeks |
| Prior2weeksHF | Heart Failure in prior 2 weeks |
| PriorCABG | Prior history of CABG |
| PriorCardiacArrest | Prior history of Cardiac Arrest |
| PriorCardioShock | Prior history of Cardiogenic Shock |
| PriorCVD | Prior history of cerebrovascular disease |
| PriorHF | Prior history of heart failure |
| PriorMI | Prior history of myocardial infarction |
| PriorPAD | Prior history of peripheral arterial disease |
| PriorPCI | Prior history of PCI |
| RaceAmIndian | Patient Race |
| RaceAsian | Patient Race |
| RaceBlack | Patient Race |
| RaceNatHaw | Patient Race |
| RaceWhite | Patient Race |
| RENFAIL | Binary variable, 1 if GFR < 30 or on Current Dialysis, indicates Renal Failure |
| SHOCKPCIS | PCI Status AND DCarShock, 1 if DCARSHOCK == 1 AND PCI Status == Salvage, 2 if DCARSHOCK == 1 OR PCI Status == Salvage, 3 if DCARSHOCK == 2 OR DCARSHOCK == 3, 4 if PCI Status == Emergency, 5 if PCI Status == Urgent, 6 Otherwise |
| SHOCKPCIS1 | if ShockPCIS == 1 |
| SHOCKPCIS2 | if ShockPCIS == 2 |
| SHOCKPCIS3 | if ShockPCIS == 3 |
| SHOCKPCIS4 | if ShockPCIS == 4 |
| SHOCKPCIS5 | if ShockPCIS == 5 |
| SHOCKPCIS6 | if ShockPCIS == 6 |
| Smoker | Smoker |
| STEMI | STEMI |
| STEMIFirstNoted | Stemi First Noted |
| ThromTherapy | Thrombolytics Given |
| ValveSurgery | Prior Valve Surgery |
| Weight | Patient Weight |
| PreOpMed4 | Aspirin |
| CardiacTransplant | Prior Cardiac Transplant |
| PeriopEval | Pre-operative evaluation before NCS |
| PatientTransPCI | Transferred in for immediate PCI |
| PCIndication_1 | Immediate PCI for STEMI |
| PCIndication_2 | PCI for STEMI (unstable) |
| PCIndication_3 | PCI for STEMI (stable) |
| PCIndication_4 | PCI for STEMI (stable after thrombolysis) |
| PCIndication_5 | Rescue PCI for STEMI |
| PCIndication_6 | PCI for high risk Non-STEMI |
| PCIndication_7 | Staged PCI |
| PCIndication_8 | Other |
| **Stage 2** |  |
| Femoral | Femoral Access |
| Radial | Radial Access |
| **Stage 3** |  |
| CIRCGStenosis | CIRC, Oms, LPDA, LPL Graft Stenosis % |
| CIRCStenosis | CIRC, Oms, LPDA, LPL Stenosis % |
| DCathStatus | Diagnostic Cath Status |
| DCathTreatment | Diagnostic Cath Treatment Recommendation Composite |
| DiagCorAngio | Diagnostic Coronary Angiography Procedure Performed |
| DiagnosticCath | Patient had Diagnostic Cath |
| DissectionSeg | Significant Dissection |
| Dominance_1 | Dominance: Left |
| Dominance_2 | Dominance: Right |
| Dominance_3 | Dominance: Co-dominant |
| LeftHeartCath | Left Heart Cath Procedure |
| LMStenosis | Left Main Stenosis % |
| MidDistalLADGStenosis | Mid/Distal LAD, Diag Graft Stenosis % |
| MidDistalLADStenosis | Mid/Distal LAD, Diagnosis Branch Stenosis % |
| PerfSeg | Indicates if angiographic or clinical evidence of perforation was observed |
| ProxLADGtStenosis | Proximal LAD Graft Stenosis % |
| ProxLADStenosis | Proximal LAD Stenosis % |
| RamusGStenosis | Ramus Graft Stenosis % |
| RamusStenosis | Ramus Stenosis % |
| RCAGStenosis | RCA, RPDA, RPL, AM Graft Stensosis % |
| RCAStenosis | RCA, RPDA, RPL, AM Stensosis % |
| PreProcTIMI | Pre Procedure TIMI Flow |
| PreTIMI | 1 - PreProc TIMI Flow |
| PRETIMINO | if PreTIMI == 0 |
| PreviousStent | Previously Treated with Stent |
| PrevTreatedLesion | Indicate if the lesion has been treated before in the current or a prior episode of care |
| StenosisPriorTreat | Stenosis Immediately Prior to Treatment |
| THROM | Previous Lesion Time check and In Thrombosis |
| Thrombus | Thrombus Present |
| **Stage 4** |  |
| Fondaparinux |  |
| Prasugrel |  |
| Low Molecular Weight Heparin |  |
| Ticagrelor |  |
| Unfractionated Heparin |  |
| BivalRudin |  |
| Direct Thrombin Inhibitor |  |
| GP llb/llla (any) |  |
| Clopidogrel |  |
| Ticlopidine |  |
| **Stage 5** |  |
| BifurcationLesion | Indicate if the lesion is at a significant bifurcation, trifurcation, or more complex branch point |
| ChronicOcclusion | Chronic Total Occlusion |
| CTO | Stenosis Prior to Treatment and Chronic Occlusion |
| Culprit | If Culprit Artery == 1 |
| CulpritArtery | Culprit Lesion |
| FFR | Fractional Flow Reserve |
| FFRatio | Fractional Flow Reserve Ratio |
| FluroTime | Indicates guidewire successfully crossed the lesion |
| GuidewireLesion | Indicate if the lesion is at a significant bifurcation, trifurcation, or more complex branch point |
| IABP | Patient Required Use of Intra Aortic Balloon Pump |
| IABPTiming | When IABP Placed (Start, during and prior to PCI, after PCI) |
| ICDEV_Atherectomy | IC Dev Atherectomy |
| ICDEV_Balloon | IC Device Balloon |
| ICDEV_Bare_Metal_Stent | IC Device Bare Metal Stent |
| ICDEV_Brachy_Therapy | IC Dev Brachy Therapy |
| ICDEV_Chronic_Total_Occlusion | IC Dev Crhonic Total Occlusion |
| ICDEV_Coated_Stent | IC Dev Coated Stent |
| ICDEV_Covered_Stent | IC Dev Covered Stent |
| ICDEV_Cutting_Balloon | IC Dev Cutting Balloon |
| ICDEV_Drug_Eluting_Stent | IC Drug Eluting Stent |
| ICDEV_Embolic_Protection | IC Dev Embolic Protection |
| ICDEV_Extraction_Catheter | IC Dev Extraction Catheter |
| ICDEV_Laser | IC Device laser |
| ICDEV_Other | IC Dev Other |
| ICDEV_Thrombectomy | IC Device Thrombectomy |
| InRestenosis | In-Stent Restenosis |
| InThrombosis | In-Stent Thrombosis |
| IVUS | Intravascular Ultrasound |
| LesionCounter.y | Lesion Counter from LSEGM |
| LesionGraft | Not in graft, vein, LIMA graft, other artery |
| LesionLength | Lesion Length |
| LesonComplexty | Lesion Complexity : 7185 |
| LESSCAI | Based on Stenosis Prior to Treatment and Lesion complexity |
| LESSCAI23 | LESSCAI is 2 or 3 |
| LocGraft_1 | Aortic |
| LocGraft_2 | Body |
| LocGraft_3 | Distal |
| LSDEV_Atherectomy | LS Dev Atherectomy |
| LSDEV_Balloon | LS Device - Balloon |
| LSDEV_Bare_Metal_Stent | LSDEV Bare Metal Stent |
| LSDEV_Brachy_Therapy | LS Dev Brachy Therapy |
| LSDEV_Chronic_Total_Occlusion | LSDev Chronic Total Occlusion |
| LSDEV_Coated_Stent | LS Dev Coated Stent |
| LSDEV_Covered_Stent | LS Dev Covered Stent |
| LSDEV_Cutting_Balloon | LS Dev Cutting Balloon |
| LSDEV_Drug_Eluting_Stent | LS Device - Drug Eluting Stent |
| LSDEV_Embolic_Protection | LS Dev Embolic Protection |
| LSDEV_Extraction_Catheter | LS Dev Extraction Catheter |
| LSDEV_Laser | LS Dev laser |
| LSDEV_Other | LS Dev Other |
| LSDEV_Thrombectomy | LS Dev Thrombectomy |
| MVSupport | Other Mechanical Ventricular Support |
| MVSupportTiming | Start of procedure, during or prior to pci, after PCI has begun |
| NEWSEQ | Based upon Segment ID (Left Main, pLAD, or pRCA, mLad, Pcirc, or none) |
| NVD | Categorical Variable on LMStenosis, RCASTenosis, ProxLADStenosis, MidDistalLADStenosis, CIRCStenosis, RamusStenosis |
| NVD23 | if NVD == 2 or 3, or, if LMSTensosis > 50 |
| OtherProcedure | Patient has other procedure in conjunction w Dx Cath or PCI) |
| PCICardioShock | Cardiogenic Shock at Start of PCI |
| PCIDelayReason_1 | Difficult vascular access |
| PCIDelayReason_2 | Cardiac arrest and/or need for intubation before PCI |
| PCIDelayReason_3 | Patient delays in providing consent for the procedure |
| PCIDelayReason_4 | Difficulty crossing the culprit lesion during the PCI procedure |
| PCIDelayReason_5 | Other |
| PCIDelayReason_6 | None |
| SegmentID_1 | Based upon NCDR Definition of Segment Number #7105 – CathPCI V4.4 |
| SegmentID_10 | Based upon NCDR Definition of Segment Number #7105 – CathPCI V4.4 |
| SegmentID_11 | Based upon NCDR Definition of Segment Number #7105 – CathPCI V4.4 |
| SegmentID_12 | Based upon NCDR Definition of Segment Number #7105 – CathPCI V4.4 |
| SegmentID_13 | Based upon NCDR Definition of Segment Number #7105 – CathPCI V4.4 |
| SegmentID_14 | Based upon NCDR Definition of Segment Number #7105 – CathPCI V4.4 |
| SegmentID_15 | Based upon NCDR Definition of Segment Number #7105 – CathPCI V4.4 |
| SegmentID_15a | Based upon NCDR Definition of Segment Number #7105 – CathPCI V4.4 |
| SegmentID_16 | Based upon NCDR Definition of Segment Number #7105 – CathPCI V4.4 |
| SegmentID_16a | Based upon NCDR Definition of Segment Number #7105 – CathPCI V4.4 |
| SegmentID_17 | Based upon NCDR Definition of Segment Number #7105 – CathPCI V4.4 |
| SegmentID_18 | Based upon NCDR Definition of Segment Number #7105 – CathPCI V4.4 |
| SegmentID_19 | Based upon NCDR Definition of Segment Number #7105 – CathPCI V4.4 |
| SegmentID_19a | Based upon NCDR Definition of Segment Number #7105 – CathPCI V4.4 |
| SegmentID_2 | Based upon NCDR Definition of Segment Number #7105 – CathPCI V4.4 |
| SegmentID_20 | Based upon NCDR Definition of Segment Number #7105 – CathPCI V4.4 |
| SegmentID_20a | Based upon NCDR Definition of Segment Number #7105 – CathPCI V4.4 |
| SegmentID_21 | Based upon NCDR Definition of Segment Number #7105 – CathPCI V4.4 |
| SegmentID_21a | Based upon NCDR Definition of Segment Number #7105 – CathPCI V4.4 |
| SegmentID_22 | Based upon NCDR Definition of Segment Number #7105 – CathPCI V4.4 |
| SegmentID_22a | Based upon NCDR Definition of Segment Number #7105 – CathPCI V4.4 |
| SegmentID_23 | Based upon NCDR Definition of Segment Number #7105 – CathPCI V4.4 |
| SegmentID_24 | Based upon NCDR Definition of Segment Number #7105 – CathPCI V4.4 |
| SegmentID_25 | Based upon NCDR Definition of Segment Number #7105 – CathPCI V4.4 |
| SegmentID_26 | Based upon NCDR Definition of Segment Number #7105 – CathPCI V4.4 |
| SegmentID_27 | Based upon NCDR Definition of Segment Number #7105 – CathPCI V4.4 |
| SegmentID_28 | Based upon NCDR Definition of Segment Number #7105 – CathPCI V4.4 |
| SegmentID_28a | Based upon NCDR Definition of Segment Number #7105 – CathPCI V4.4 |
| SegmentID_29 | Based upon NCDR Definition of Segment Number #7105 – CathPCI V4.4 |
| SegmentID_29a | Based upon NCDR Definition of Segment Number #7105 – CathPCI V4.4 |
| SegmentID_3 | Based upon NCDR Definition of Segment Number #7105 – CathPCI V4.4 |
| SegmentID_4 | Based upon NCDR Definition of Segment Number #7105 – CathPCI V4.4 |
| SegmentID_5 | Based upon NCDR Definition of Segment Number #7105 – CathPCI V4.4 |
| SegmentID_6 | Based upon NCDR Definition of Segment Number #7105 – CathPCI V4.4 |
| SegmentID_7 | Based upon NCDR Definition of Segment Number #7105 – CathPCI V4.4 |
| SegmentID_8 | Based upon NCDR Definition of Segment Number #7105 – CathPCI V4.4 |
| SegmentID_9 | Based upon NCDR Definition of Segment Number #7105 – CathPCI V4.4 |
| StentType | Drug eluting stent, non-drug eluting stent |
| ContrastVol | Contrast volume |
| **Stage 6** |  |
| closure_Other | Closure Device: Other |
| closure_Manual_com | Closure Device: Manual Compression |
| closure_Suture | Closure Device: Suture |
| closure_Staple | Closure Device: Staple |
| closure_Sealant | Closure Device: Sealant |
| closure_Patch | Closure Device: Patch |
| closure_Mechanical | Closure Device: Mechanical |
| total_count_closure | Count of Closure Devices |
| closure_None | Closure Device: None |
