# Supplementary material for: Towards a dynamic model to estimate evolving risk of major bleeding after percutaneous coronary intervention
Source: PLOS Digit Health. 2025 Jun 25;4(6):e0000906. doi: 10.1371/journal.pdig.0000906 (PMC12193038; doi:10.1371/journal.pdig.0000906)
Supplement: S1 Text — (DOCX) [file pdig.0000906.s003.docx]

*Multicollinearity Analysis*

The primary purpose of this manuscript was to take the data available to the prior models and show that if you add data over time, the staged analysis shows changes in risk stratification. However, one might look at the data and notice both continuous variables (such as pre-procedure hemoglobin) and derived, indicator daughter variables (e.g. pre-procedure hemoglobin less than or equal to 13). An advantage of XGBoost is that the divide and conquer approach it takes towards building weak learners allows it to find non-linear relationships between the best predictive features given the particular subset of participant data each tree is being trained on. This has an advantage where a traditional logistic regression would not, being able to handle many similar, collinear variables such as these indicators or missing/not drawn features. However, to be sure that these are not adversely impacting modeling, we conducted an analysis where we removed the collinear features that may cause confusion from **S1 Table**, highlighted, and re-ran our analysis. **S3 Fig and S4 Fig** show the AUROC and AUPRC calculations. As noted, these do similarly but slightly worse. This is likely because the continuous and categorical parent variables are stronger predictors over the entire population, but given the depths of the trees and the number of weak learners built to make the strong XGBoost model, it is likely that the interaction between the daughter variables and some of the other features are clearer predictors for some subsets of participants in the dataset. This drop in performance shows that clinical expertise injected into feature creation and usage can still support a number of these models. Similarly, **S9 Fig, S10 Fig, S11 Fig, S12 Fig, S13 Fig, and S14 Fig** show the SHAP explainer plots for each model stage. The primary predictors remain consistent. An interesting finding is the inclusion of Pre-procedure hemoglobin and its two daughter dichotomous indicator variables in the primary analysis, and just pre-procedure hemoglobin in this analysis. As can be seen in these SHAP plots, the risk that this value provides does not monotonically increase or decrease with the hemoglobin value. This is why in the primary analysis we see both Pre-Procedure Hemoglobin > 13 and Pre-Procedure Hemoglobin <= 13 having potentially increasing risk. Unfortunately, SHAP is not able to show which of these features interact with each within the depths of the learned trees or coverage over the trees that would help further illustrate interpretability. Ultimately, a parsimonious model would need to be built with available predictors in an interpretable fashion to drive clinical understanding, but given the registry data available here in a retrospective analysis, this work focused on further highlighting need to think about time-varying data when conducting risk stratification.
