# Supplementary material for: Towards a dynamic model to estimate evolving risk of major bleeding after percutaneous coronary intervention
Source: PLOS Digit Health. 2025 Jun 25;4(6):e0000906. doi: 10.1371/journal.pdig.0000906 (PMC12193038; doi:10.1371/journal.pdig.0000906)
Supplement: S2 Table — (DOCX) [file pdig.0000906.s005.docx]

**S2 Table** Comparison of model performances for bleeding prediction by subgroup on access site.

| **Model** | **Overall AUROC** | **Femoral AUROC** | **Radial AUROC** |
| --- | --- | --- | --- |
| **Presentation** | 0.812 (0.812-0.812) | 0.804(0.804,0.804) | 0.822(0.822,0.822) |
| **+ Access Site** | 0.817 (0.817-0.817) | 0.804(0.803,0.804) | 0.825(0.825,0.825) |
| **+ Cath Lab** | 0.825 (0.825-0.825) | 0.812(0.812,0.812) | 0.834(0.834,0.835) |
| **+ Medication Decision** | 0.832 (0.832-0.832) | 0.820(0.820,0.820) | 0.840(0.839,0.840) |
| **+ PCI Variables** | 0.844 (0.844-0.845) | 0.834(0.834,0.834) | 0.850(0.850,0.851) |
| **+ Closure Decision** | 0.845 (0.845-0.845) | 0.835(0.835,0.835) | 0.851(0.850,0.851) |
