# Supplementary material for: Towards a dynamic model to estimate evolving risk of major bleeding after percutaneous coronary intervention
Source: PLOS Digit Health. 2025 Jun 25;4(6):e0000906. doi: 10.1371/journal.pdig.0000906 (PMC12193038; doi:10.1371/journal.pdig.0000906)
Supplement: S2 Text — (DOCX) [file pdig.0000906.s006.docx]

*Comparing Results by Subgroup (Femoral vs. Radial)*

Knowing that radial and femoral access choice lead to different findings, we also conducted a subgroup analysis in which we evaluated model performance on each subgroup. As noted in **S2 Table**, we find that radial access expectedly performs better, likely because bleeds are less frequent and those that happen are clearer based upon key risk factors. However, what is of note here is that both femoral and radial see increased AUROC, with increased risk factor association with outcome over the stages and therefore the findings of the main models are consistent across the key subgroups here.
