# Supplementary material for: Towards a dynamic model to estimate evolving risk of major bleeding after percutaneous coronary intervention
Source: PLOS Digit Health. 2025 Jun 25;4(6):e0000906. doi: 10.1371/journal.pdig.0000906 (PMC12193038; doi:10.1371/journal.pdig.0000906)

**S4 Fig.** Precision Recall Curves for each staged model with a representative fold taken at random for each stage of the model and each 5-fold cross-validation for the collinearity analysis
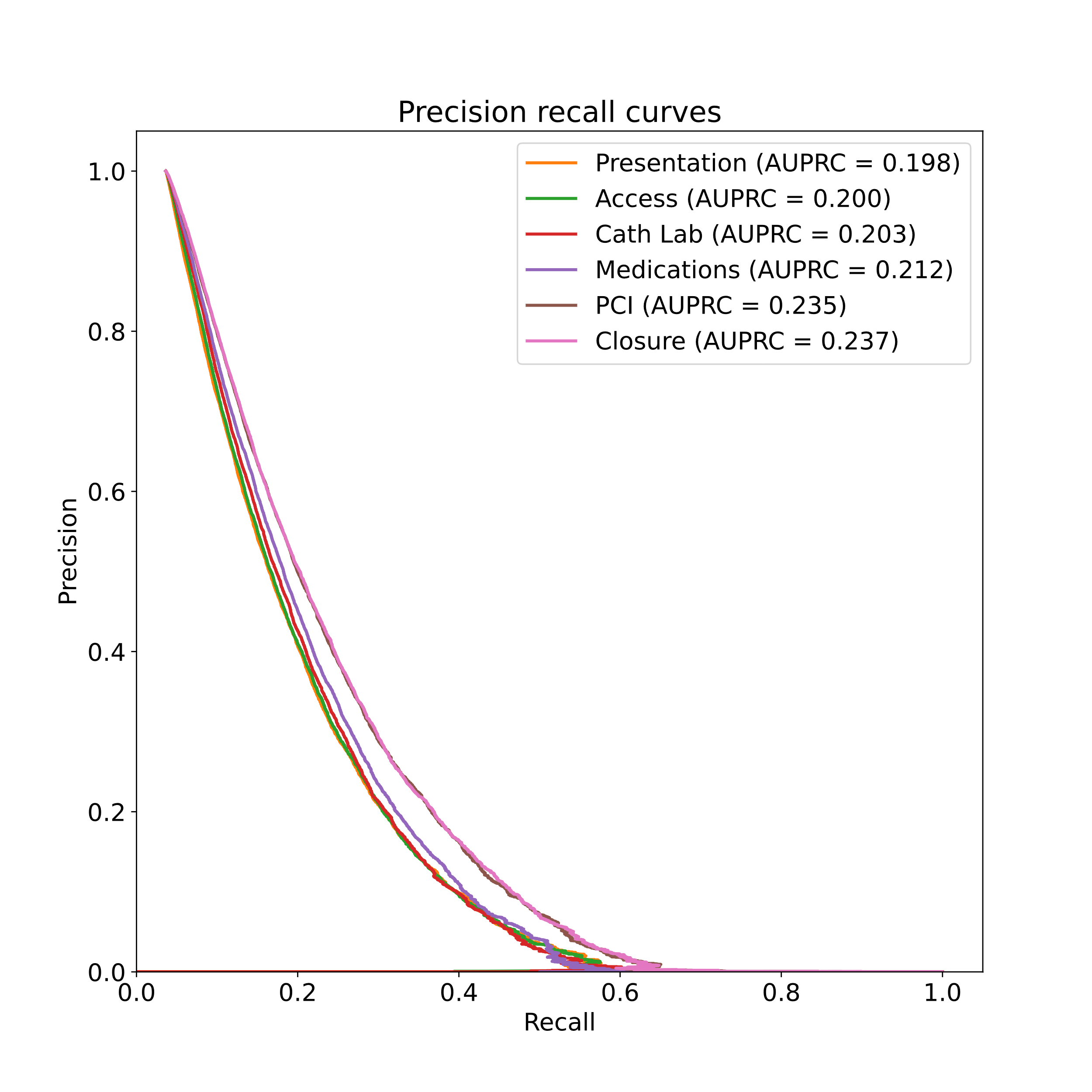

Supplement: S3 Fig — (DOCX) [file pdig.0000906.s007.docx]
