# Supplementary material for: Towards a dynamic model to estimate evolving risk of major bleeding after percutaneous coronary intervention
Source: PLOS Digit Health. 2025 Jun 25;4(6):e0000906. doi: 10.1371/journal.pdig.0000906 (PMC12193038; doi:10.1371/journal.pdig.0000906)
Supplement: S3 Table — Top value in each cell is number of patients classified into that risk bin by the two respective models. The bottom value in each cell indicates the actual bleeding rate of all patients within that cell. S3 Table shows shift tables before and after each decision, while Table 3 shows a shift table from the initial to final model. The earlier model is displayed left to right, while the later model is displayed top to bottom. The top number in each cell represents the number of patients assigned to that risk bin by each model. The bottom number in each cell is the overall bleeding rate of all patients in that cell. NaN represents that no patients were in that combination of bins. (DOCX) [file pdig.0000906.s008.docx]

**S3 Table.** Shift tables following each decision point. Top value in each cell is number of patients classified into that risk bin by the two respective models. The bottom value in each cell indicates the actual bleeding rate of all patients within that cell. **S3 Table** shows shift tables before and after each decision, while **Table 3** shows a shift table from the initial to final model. The earlier model is displayed left to right, while the later model is displayed top to bottom. The top number in each cell represents the number of patients assigned to that risk bin by each model. The bottom number in each cell is the overall bleeding rate of all patients in that cell. NaN represents that no patients were in that combination of bins.

| **Initial vs After Access Site Decision** | | | | |
| --- | --- | --- | --- | --- |
|  | **Model 1** | | | |
|  | **<1%** | **1-4%** | **>4%** | **All** |
| **Model 2** | **Patients, N** | **Patients, N** | **Patients, N** | **Patients, N** |
|  | **Observed Rate** | **Observed Rate** | **Observed Rate** | **Observed Rate** |
| <1% | 114,641 | 33,129 |  | 147,770 |
|  | 0.57% | 0.54% | NaN | 0.56% |
| 1-4% | 9,071 | 230,891 | 14,582 | 254,544 |
|  | 0.99% | 1.69% | 2.54% | 1.72% |
| >4% |  | 6,465 | 145,583 | 152,048 |
|  | NaN | 3.11% | 10.14% | 9.84% |
| All | 123,712 | 270,485 | 160,165 | 554,362 |
|  | 0.60% | 1.58% | 9.45% | 3.64% |
| **In Cath Lab Before vs After Medication Decisions** | | | | |
|  | **Model 3** | | | |
|  | **<1%** | **1-4%** | **>4%** | **All** |
| **Model 4** | **Patients, N** | **Patients, N** | **Patients, N** | **Patients, N** |
|  | **Observed Rate** | **Observed Rate** | **Observed Rate** | **Observed Rate** |
| <1% | 140,103 | 32,764 |  | 172,867 |
|  | 0.43% | 0.64% | NaN | 0.47% |
| 1-4% | 11,244 | 207,541 | 21,612 | 240,397 |
|  | 1.20% | 1.67% | 3.24% | 1.79% |
| >4% |  | 11,448 | 129,650 | 141,098 |
|  | NaN | 4.37% | 11.22% | 10.67% |
| All | 151,347 | 251,753 | 151,262 | 554,362 |
|  | 0.49% | 1.66% | 10.08% | 3.64% |
| **Post PCI Before vs After Closure Decision** | | | | |
|  | **Model 5** | | | |
|  | **<1%** | **1-4%** | **>4%** | **All** |
| **Model 6** | **Patients, N** | **Patients, N** | **Patients, N** | **Patients, N** |
|  | **Observed Rate** | **Observed Rate** | **Observed Rate** | **Observed Rate** |
| <1% | 178,911 | 12,095 |  | 191,006 |
|  | 0.43% | 0.54% | NaN | 0.44% |
| 1-4% | 8,561 | 213,367 | 7,268 | 229,196 |
|  | 0.81% | 1.73% | 3.56% | 1.76% |
| >4% |  | 5,703 | 128,457 | 134,160 |
|  | NaN | 3.30% | 11.76% | 11.40% |
| All | 187,472 | 231,165 | 135,725 | 554,362 |
|  | 0.45% | 1.71% | 11.32% | 3.64% |
