# Supplementary material for: Towards a dynamic model to estimate evolving risk of major bleeding after percutaneous coronary intervention
Source: PLOS Digit Health. 2025 Jun 25;4(6):e0000906. doi: 10.1371/journal.pdig.0000906 (PMC12193038; doi:10.1371/journal.pdig.0000906)
Supplement: S3 Text — (DOCX) [file pdig.0000906.s009.docx]

*Decision 1: Access Site (Model 2)*

The first decision point in this model is the choice of arterial access site: femoral or radial. When accounting for this decision, the AUROC improved to 0.817 and the AUPRC improved to 0.204. The Brier skill score improved to 0.091, the Brier reliability improved to 2.0E-4. The SHAP plot shows that femoral access is the ninth most informative variable in Model 2 (**S5 Fig**). The inclusion of seemingly mutually exclusive variables (higher hemoglobin vs. lower hemoglobin) both being important is a function of different decision trees. Procedures performed via femoral access had a slightly increased rate of bleeding, while those performed via a radial access site had a variably decreased rate of bleeding. The fact that these procedures have similar SHAP values indicates that the model assigns similar risk to procedures with femoral access.

**S3 Table** presents a shift table describing patient risk categories^19^. Among 123,712 patients classified as low (<1%) risk of bleeding by the clinical presentation model, 9,071 (7.3%) were reclassified as medium (1-4%) risk of bleeding by the model incorporating access site (**S3 Table**). Among those reclassified, 0.99% experienced a bleeding event. Among 270,485 patients classified as medium risk of bleeding by the initial model, 33,129 (12.2%) were reclassified as low risk by the subsequent model, while 6,465 (2.4%) were reclassified as high (>4%) risk. Of the 33,129 patients reclassified as low risk, 0.5% experienced a bleeding event, while of the 6,465 patients reclassified as high risk, 3.1% exhibited a bleeding event. Among 160,165 patients classified as high risk of bleeding by the initial model, 14,582 (9.1%) were reclassified as medium risk. Among those patients, 2.5% experienced a bleeding event.

*Stage 2: Cardiac Catheterization Laboratory (Model 3)*

The cardiac catheterization laboratory model uses information available after performing a diagnostic cardiac catheterization, but prior to initiation of PCI and choice of peri-procedural medications. In this model, bleeding prediction improved with an AUROC of 0.825 and an AUPRC of 0.208. The Brier skill score improved to 0.094, the Brier reliability worsened to 2.1E-4, and the Brier resolution improved to 3.5E-3. Of features added in this model, the SHAP plot shows that predictions are highly influenced by the presence of thrombus in a coronary lesion (13^th^ most informative variable) and pre-procedure Thrombolysis in Myocardial Infarction (TIMI) flow (14^th^ most informative variable) (**S6 Fig**).

*Decision 2: Intra-Procedure Medication (Model 4)*

The next decision point is the choice of intra-procedural antiplatelet and anticoagulant agents. Following inclusion of these variables, the model performance increases to an AUROC of 0.832 and an AUPRC of 0.217. The Brier skill score improved to 0.102, the Brier reliability improved to 1.4E-4, and the Brier resolution improved to 3.7E-3. Of features added in this model the SHAP plot shows that use of glycoprotein IIb/IIIa inhibitors was strongly associated with an increased risk of bleeding (4^th^ most informative variable), while unfractionated heparin was less strongly associated with an increased risk of bleeding (14^th^ most informative variable) (**S7 Fig**).

Among 151,347 patients classified as low risk of bleeding by the cardiac catheterization lab model (Model 3), 11,244 (7.6%) were reclassified as moderate risk by the model incorporating medication choices (Model 4), (**S3 Table**). Among those 11,244 reclassified patients, 1.2% experienced bleeding events, reflecting that these reclassifications improved model calibration. Among 251,753 patients classified as moderate risk of bleeding by Model 3, 32,764 (13.0%) were reclassified as low risk by Model 4, while 11,448 (4.5%) were reclassified as high risk. Among the 32,764 patients reclassified as low risk 0.6% experienced bleeding events, among the 11,448 patients reclassified as high risk 4.4% experienced bleeding events, also reflecting that the reclassifications improved calibration. Among 151,262 patients classified as high risk of bleeding by Model 3, there were 21,612 (14.3%) patients who were reclassified as moderate risk by Model 4. Among those patients, 3.2% experienced a bleeding event.

*Stage 3: PCI (Model 5)*

The post-PCI model uses all information through PCI but prior to choice of closure method. This model improves upon the performance of prior models, with an AUROC of 0.844 and AUPRC of 0.241. The Brier skill score improved to 0.118, the Brier reliability improved to 1.1E-4, and the Brier resolution improved to 4.2E-3. The new features introduced in this model that were most associated with increased bleeding risk are proxies of PCI complexity and duration, including fluoroscopy time (10^th^ most informative variable) and contrast volume (20^th^ most important variable) (**S8 Fig**).

*Decision 3: Closure Method (Model 6)*

Among 187,472 patients classified as low risk of bleeding by the post-PCI model (Model 5), 8,561 (4.6%) were reclassified as moderate risk by the model incorporating closure decision (Model 6) (Table 3C). Bleeding events occurred in 0.8% of those patients. Among 231,165 patients classified as moderate risk of bleeding by Model 5, 12,095 (5.2%) were reclassified as low risk by Model 6, while 5,703 (2.5%) were reclassified as high risk. While the patients reclassified as low risk had an appropriately low occurrence of bleeding (0.54%), those patients reclassified to high risk had a moderate aggregated occurrence of bleeding (3.30%). Among 135,725 patients classified as high risk of bleeding by Model 5, there were 7,268 (5.4%) patients who were reclassified as moderate risk by Model 6. Bleeding events occurred in 3.6% of the reclassified patients.

*Why XGBoost Can Report Mutually Exclusive Variables as Important*

One might initially expect variables with the same concept to have only one side show key importance. For example, when hemoglobin is dichotomized, Logistic Regression would pick one and provide a coefficient value that describes the general model performance with respect to that risk factor. However, XGBoost, in the ability to make multiple decision trees to interpret importance across all types of cases, can have some trees where the higher hemoglobin variable is a stronger determining risk factor and in others, it might select the lower hemoglobin variable. While removing these to simplify interpretation may not impact model performance, they have been left as is to highlight the varied nature of risk across all participants, and for direct comparison with prior literature.
