# Supplementary material for: Towards a dynamic model to estimate evolving risk of major bleeding after percutaneous coronary intervention
Source: PLOS Digit Health. 2025 Jun 25;4(6):e0000906. doi: 10.1371/journal.pdig.0000906 (PMC12193038; doi:10.1371/journal.pdig.0000906)

**S5 Fig.** SHAP Tree explainer for Model 2: Access Site. Procedures performed via femoral access are represented by the narrow red line to the right of the axis. In contrast, the blue points to the left of the axis represent procedures performed with radial access, and their elongated shape indicates that the radial access has a variable effect on bleeding risk, with the risk for some procedures being decreased by much more than the risk for others.


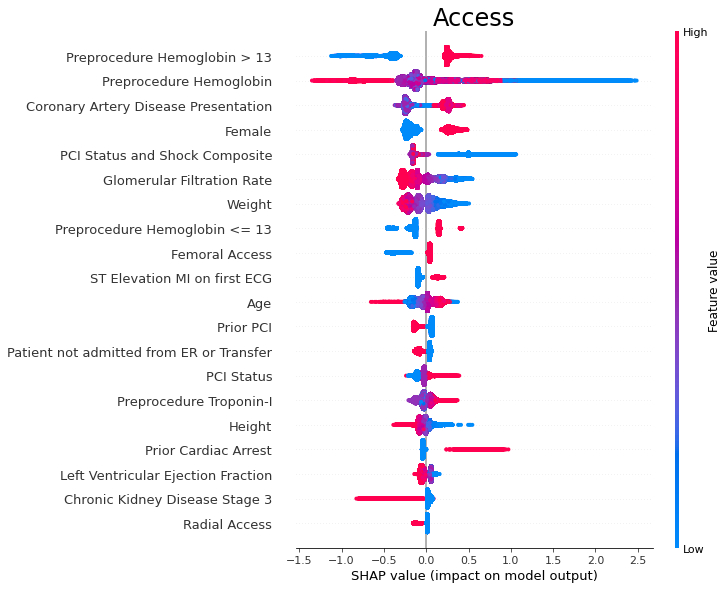

Supplement: S4 Fig — (DOCX) [file pdig.0000906.s010.docx]
