# Supplementary material for: Towards a dynamic model to estimate evolving risk of major bleeding after percutaneous coronary intervention
Source: PLOS Digit Health. 2025 Jun 25;4(6):e0000906. doi: 10.1371/journal.pdig.0000906 (PMC12193038; doi:10.1371/journal.pdig.0000906)
Supplement: S4 Table — (DOCX) [file pdig.0000906.s011.docx]

**S4 Table.** Demographic information for overall cases, and broken up into those with and without bleeds.

|  |  | Overall | Bleed | No Bleed |
| --- | --- | --- | --- | --- |
|  |  | (n=2,868,808) | (n=118,327) | (n=2,750,481) |
| Post-PCI Major Bleeds | | 118,327 (4.1%) | 118,327 (100.0%) | 0 (0.0%) |
| Demographics | |  |  |  |
|  | Age, mean (SD), y | 64.6 (12.0) | 67.3 (12.8) | 64.5 (12.0) |
|  | Men | 1,960,409 (68.3%) | 60,029 (50.7%) | 1,900,379 (69.1%) |
|  | Women | 908,399 (31.7%) | 58,298 (49.3%) | 850,102 (30.9%) |
|  | BMI, mean (SD) | 30.0 (6.4) | 28.8 (6.8) | 30.1 (6.4) |
| Cardiovascular risk factors | |  |  |  |
|  | Diabetes | 1,057,221 (36.9%) | 44,337 (37.5%) | 1,012,883 (36.8%) |
|  | Hypertension | 2,353,798 (82.1%) | 94,856 (80.2%) | 2,258,942 (82.1%) |
|  | Peripheral Vascular Disease | 339,316 (11.8%) | 17,584 (14.9%) | 321,734 (11.7%) |
|  | Chronic Kidney Disease | 861,391 (30.0%) | 53,282 (45.0%) | 808,108 (29.4%) |
| Established Coronary Artery Disease | |  |  |  |
|  | Previous PCI | 1,178,346 (41.1%) | 35,356 (29.9%) | 1,142,990 (41.6%) |
|  | Previous CABG | 510,781 (17.8%) | 17,126 (14.5%) | 493,654 (18.0%) |
| Procedural Status | |  |  |  |
|  | Elective | 1,196,485 (41.7%) | 22,630 (19.1%) | 1,173,856 (42.7%) |
|  | Urgent | 1,152,328 (40.2%) | 40,081 (33.9%) | 1,112,248 (40.4%) |
|  | Emergent | 512,404 (17.9%) | 53,747 (45.4%) | 458,656 (16.7%) |
|  | Salvage | 6,440 (0.2%) | 1,826 (1.5%) | 4,615 (0.2%) |
|  | Unknown | 1,150 (0.04%) | 43 (0.04%) | 1,108 (0.04%) |
|  | STEMI | 468,270 (16.3%) | 49,843 (42.1%) | 418,425 (15.2%) |
|  | Shock | 64,743 (2.3%) | 15,316 (12.9%) | 49,426 (1.8%) |
|  | Cardiac arrest within 24h of PCI | 49,008 (1.7%) | 10,954 (9.3%) | 38,056 (1.4%) |
|  | Preprocedural hemoglobin, median (IQR), g/dL | 13.7 (12.4-14.9) | 13.6 (11.5-15) | 13.7 (12.4-14.9) |
| Access Site |  |  |  |  |
|  | Femoral | 2,394,173 (83.5%) | 108,748 (91.9%) | 2,285,425 (83.1%) |
|  | Radial | 474,635 (16.5%) | 9,579 (8.1%) | 465,056 (16.9%) |
| Medications Used | |  |  |  |
|  | Fondaparinux | 15,816 (0.6%) | 646 (0.5%) | 15,170 (0.6%) |
|  | Prasugrel | 433,079 (15.1%) | 14,157 (12.0%) | 418,922 (15.2%) |
|  | Low Molecular Weight Heparin | 272,261 (9.5%) | 11,411 (9.6%) | 260,850 (9.5%) |
|  | Ticagrelor | 167,838 (5.9%) | 6,637 (5.6%) | 161,201 (5.9%) |
|  | Unfractionated Heparin | 1,528,882 (53.3%) | 79,097 (66.8%) | 1,449,785 (52.7%) |
|  | Bivalirudin | 1,695,225 (59.1%) | 53,244 (45.0%) | 1,641,981 (59.7%) |
|  | Direct Thrombin Inhibitor | 29,512 (1.0%) | 1,416 (1.2%) | 28,096 (1.0%) |
|  | GP llb/llla (any) | 677,865 (23.6%) | 53,786 (45.5%) | 624,079 (22.7%) |
|  | Clopidogrel | 1,988,178 (69.3%) | 79,170 (66.9%) | 1,909,008 (69.4%) |
|  | Ticlopidine | 5,895 (0.2%) | 242 (0.2%) | 5,653 (0.2%) |
| Closure Method | |  |  |  |
|  | Manual Compression | 965,618 (33.7%) | 54,835 (46.3%) | 910,783 (33.1%) |
|  | Sealant | 916,374 (31.9%) | 32,252 (27.3%) | 884,122 (32.1%) |
|  | Mechanical | 512,968 (17.9%) | 14,670 (12.4%) | 498,298 (18.1%) |
|  | Suture | 264,494 (9.2%) | 9,046 (7.6%) | 255,448 (9.3%) |
|  | Patch | 99,690 (3.5%) | 3,529 (3.0%) | 96,161 (3.5%) |
|  | Staple | 184 (0.0%) | 2 (0.0%) | 182 (0.0%) |
|  | Other | 94,818 (3.3%) | 3,154 (2.7%) | 91,664 (3.3%) |
|  | None | 14,662 (0.5%) | 839 (0.7%) | 13,823 (0.5%) |
