# Supplementary material for: Towards a dynamic model to estimate evolving risk of major bleeding after percutaneous coronary intervention
Source: PLOS Digit Health. 2025 Jun 25;4(6):e0000906. doi: 10.1371/journal.pdig.0000906 (PMC12193038; doi:10.1371/journal.pdig.0000906)
Supplement: S4 Text — (DOCX) [file pdig.0000906.s012.docx]

*Variables of Interest*

The current full existing NCDR bleeding risk model (3) uses 31 variables: 23 patient characteristics at the time of presentation and 8 characteristics related to coronary anatomy and lesion characterization. These variables included:

1. Two- or Three-vessel disease
2. STEMI
3. SCAI class II or III
4. SCAI class IV
5. Preprocedural TIMI flow grade is 0.
6. Left Main PCI
7. Prox LAD PCI
8. Subacute stent thrombosis

*Closure Devices and Radial Access*

The choice of closure device is a key indicator variable. While the choice of closure for radial access would be expected to be none, there are closure devices used with Radial Access. Without an ability to do a chart review, we take this data as is. 88% of Radial patients are coded as having a mechanical closure, 8% are coded as having manual compression and 4% as having a patch. The ability to verify the quality of the closure device data is a chief limitation of the staged-approach.

*Cleaning of data to correct missing data interpretation*

First, situations exist where a parent variable value of “no” indicates that daughter variables would not be captured (e.g., in a non-diabetic patient, no diabetic therapy is coded). Most daughter variables already had a category of missing, unknown, or other. We re-categorized the daughter variables to have a value of No/Not measured, and integrated “missing” for the few cases where the parent variable was a Yes/Measured variable and daughter variable was in fact missing.

Second, medications were categorized as no, yes, blinded (ie as a part of a clinical trial), or contraindicated. We re-categorized blinded as missing and re-categorized contraindicated as no.

Third, missing values were imputed using multiple multivariate feature imputation. Each missing feature was modeled using Bayesian ridge regressors trained in a round-robin fashion. Following imputation, binary and ordinal variables were set to the nearest allowed value. Multiple imputations were produced by sampling from the regressor models multiple times; each discrete sampling was a new overall sample from the model. This sampling was used to produce five folds of imputations.

*Why XGBoost*

XGBoost can evaluate higher order, non-linear interactions between variables automatically. This is necessary, since bleeding models based upon logistic regression selected the key variables based primarily on statistical tests between the variable and its relationship with incidence of major bleeding (3). While Logistic Regression can model with higher order, non-linear variables, these interaction terms need to be curated in advance.

*Precision and Recall Curves*

The area under the precision recall curve is directly impacted by the number of positive predictions made versus false positive or false negative estimates made, an important factor when considering cases with low event rates such as major bleeding (4.1%, **Table 1**). The precision-recall curves are plotted in **S2 Fig.**
