# Supplementary figures and images for: Towards a dynamic model to estimate evolving risk of major bleeding after percutaneous coronary intervention
Source: PLOS Digit Health. 2025 Jun 25;4(6):e0000906. doi: 10.1371/journal.pdig.0000906 (PMC12193038; doi:10.1371/journal.pdig.0000906)

**S6 Fig.** SHAP Tree explainer for Model 3: Cath Lab Visit.


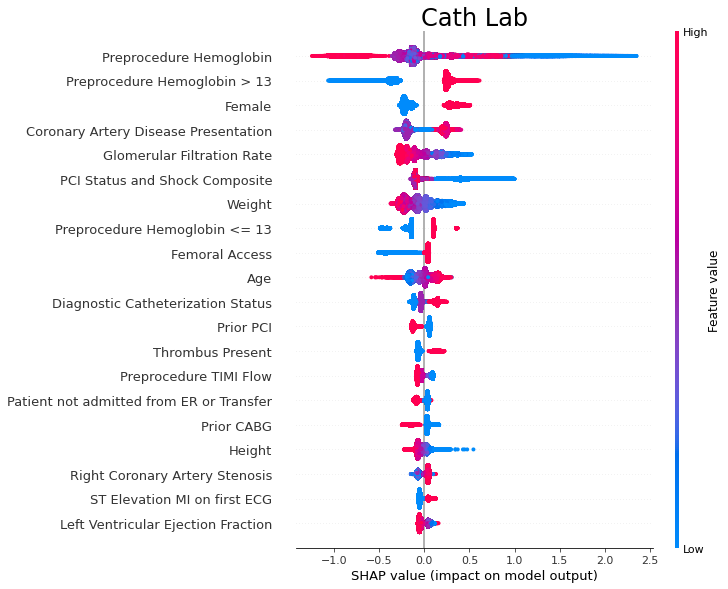

Supplement: S5 Fig — Procedures performed via femoral access are represented by the narrow red line to the right of the axis. In contrast, the blue points to the left of the axis represent procedures performed with radial access, and their elongated shape indicates that the radial access has a variable effect on bleeding risk, with the risk for some procedures being decreased by much more than the risk for others. (DOCX) [file pdig.0000906.s013.docx]

**S7 Fig.** SHAP Tree explainer for Model 4: Pre-Operative Medication Prescription


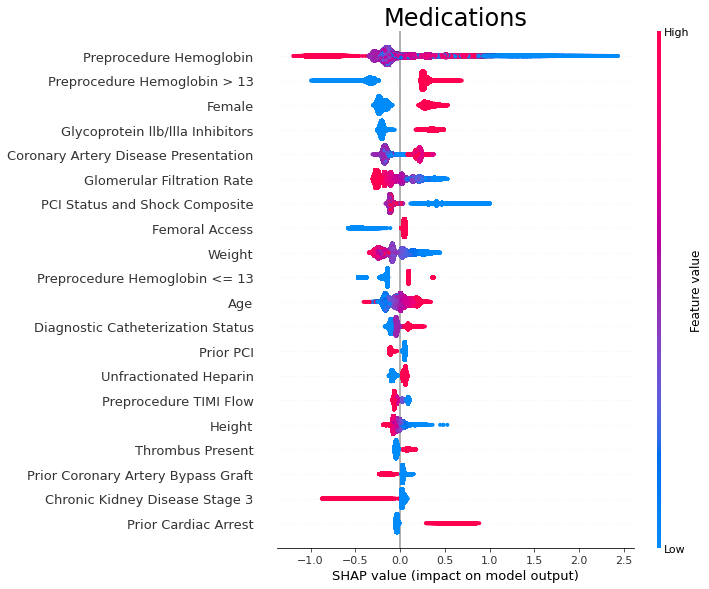

Supplement: S6 Fig — (DOCX) [file pdig.0000906.s015.docx]

**S8 Fig.** SHAP Tree explainer for Model 5: PCI


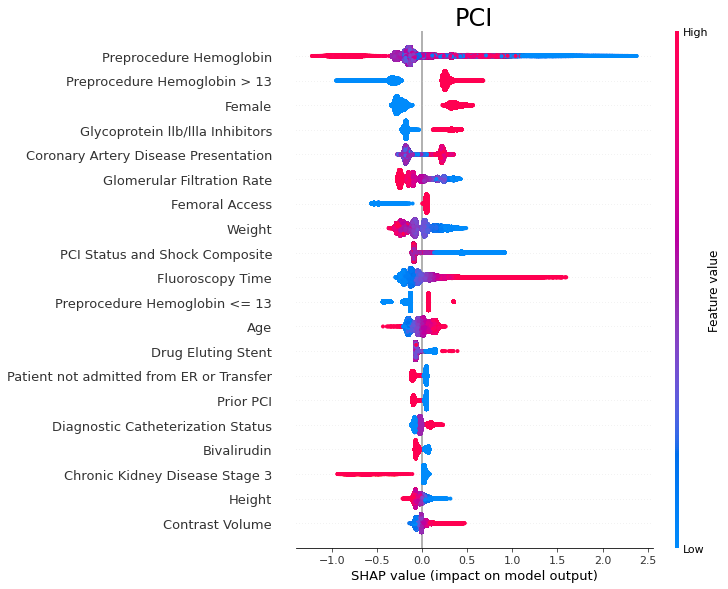

Supplement: S7 Fig — (DOCX) [file pdig.0000906.s016.docx]

**S9 Fig.** SHAP Tree explainer for Model 1: Presentation – of the multicollinearity analysis.

**
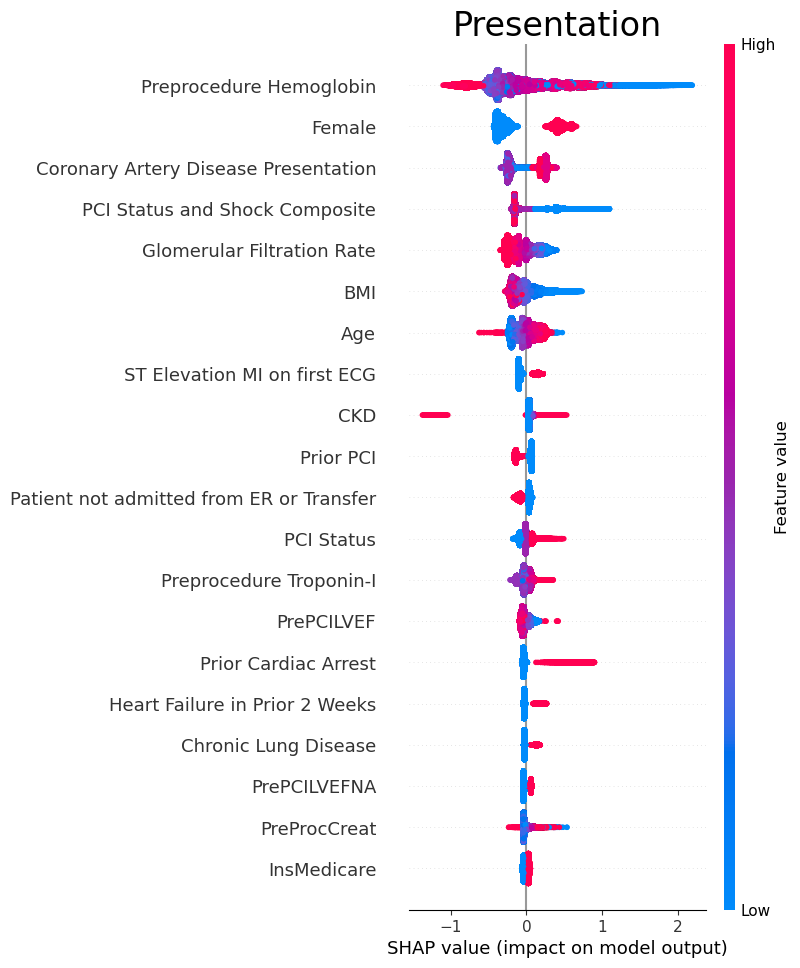
**

Supplement: S8 Fig — (DOCX) [file pdig.0000906.s017.docx]

**S10 Fig.** SHAP Tree explainer for Model 2: Access Site of the multicollinearity analysis.**
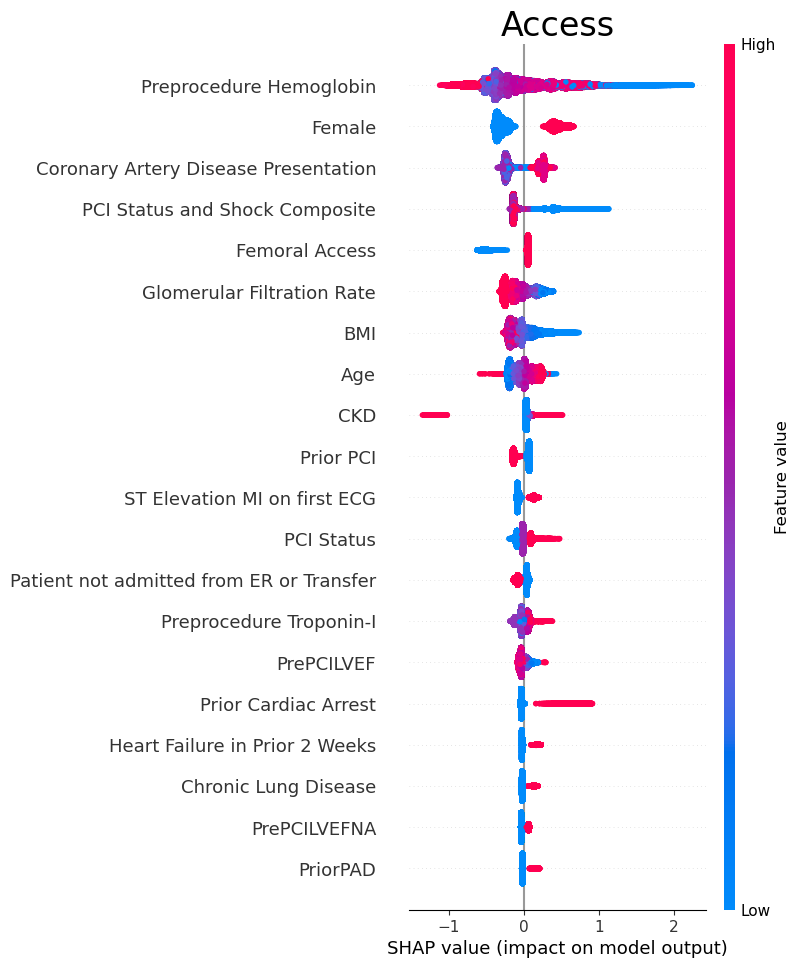
**

Supplement: S9 Fig — (DOCX) [file pdig.0000906.s018.docx]

**S11 Fig.** SHAP Tree explainer for Model 3: Cath Lab – of the multicollinearity analysis**
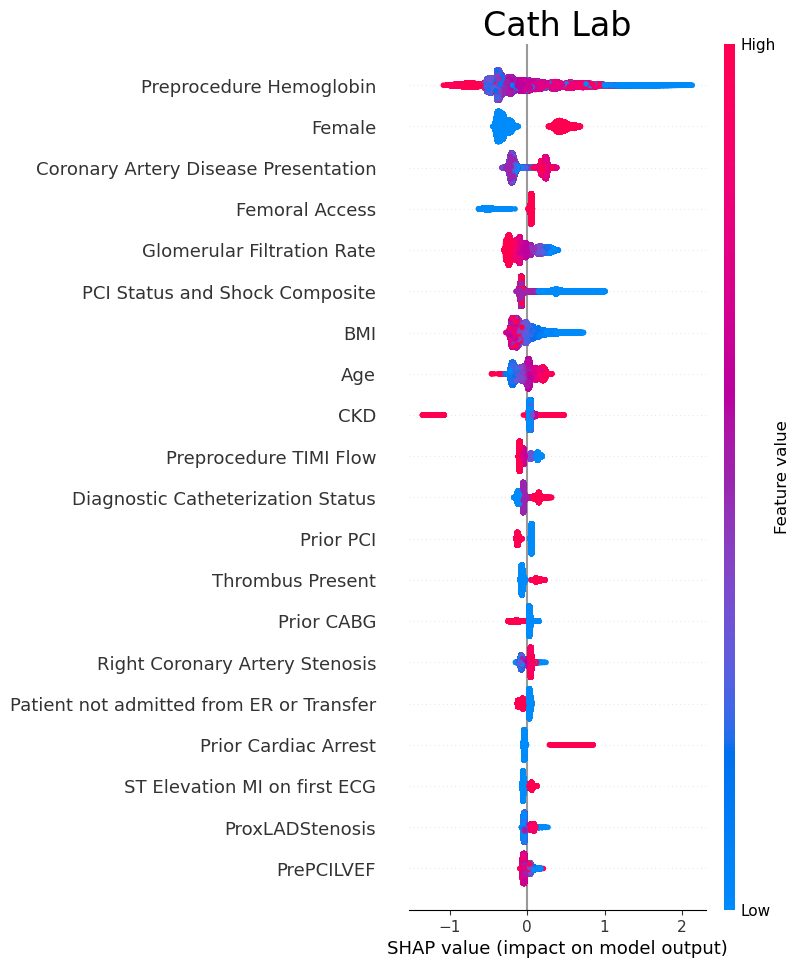
**

Supplement: S10 Fig — (DOCX) [file pdig.0000906.s019.docx]

**S12 Fig.** SHAP Tree explainer for Model 4: Medications – of the multicollinearity analysis.**
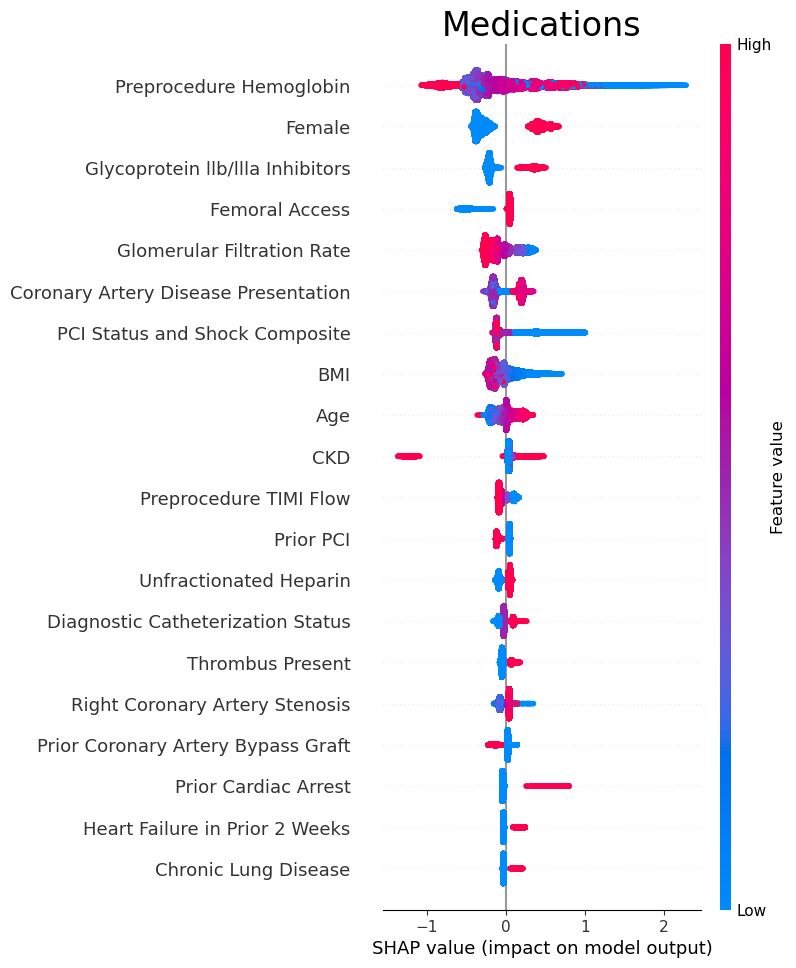
**

Supplement: S11 Fig — (DOCX) [file pdig.0000906.s020.docx]

**S13 Fig.** SHAP Tree explainer for Model 5: PCI – of the multicollinearity analysis. **
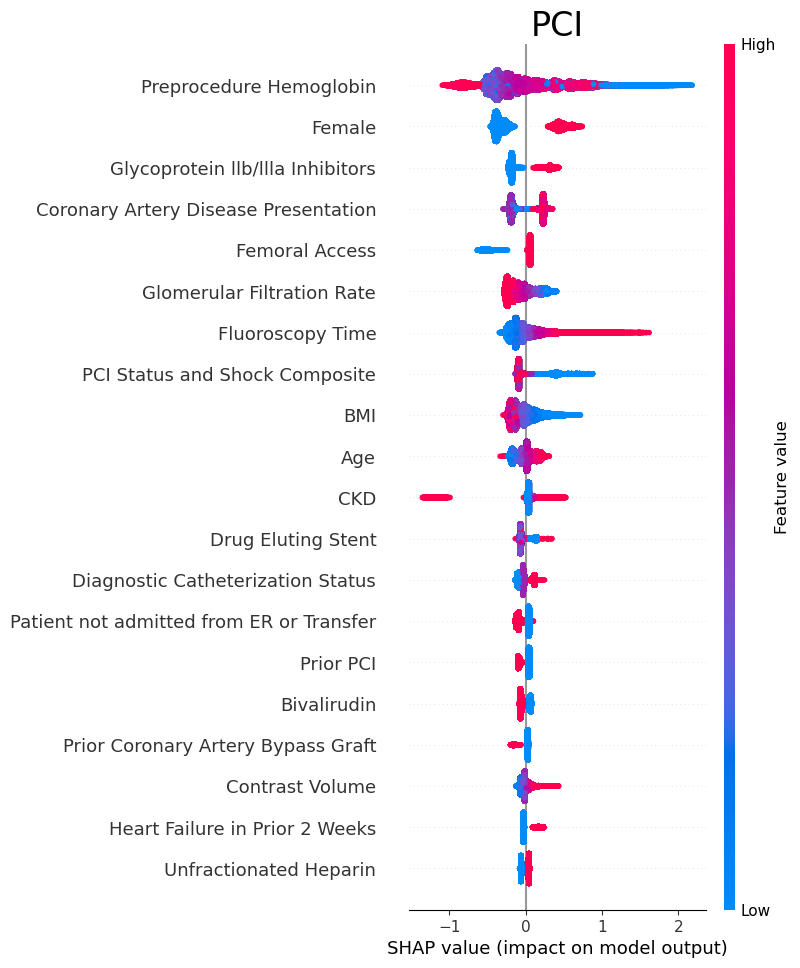
**

Supplement: S12 Fig — (DOCX) [file pdig.0000906.s021.docx]

**S14 Fig.** SHAP Tree explainer for Model 6: Closure – of the multicollinearity analysis.

**
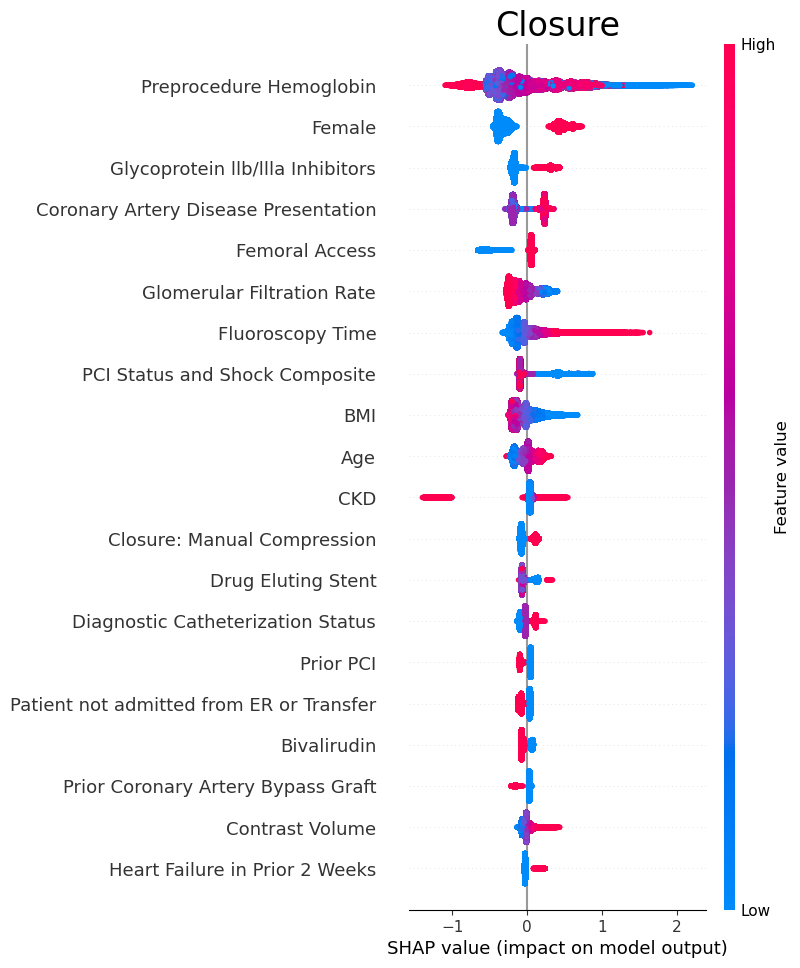
**

Supplement: S13 Fig — (DOCX) [file pdig.0000906.s022.docx]

**S17 Fig.** SHAP explainer for Case Study B.
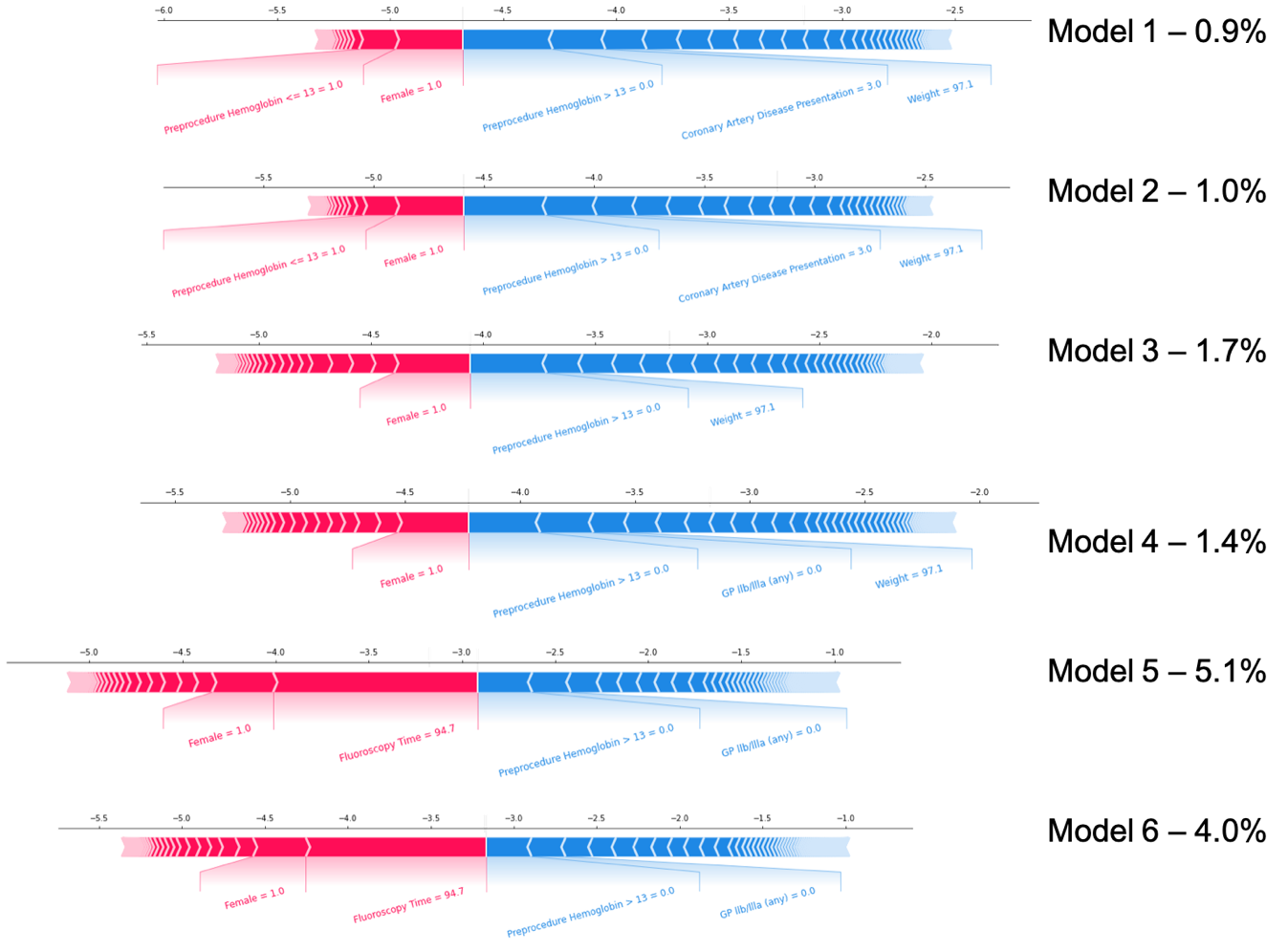

Supplement: S16 Fig — At each model stage, the prediction is created by summing each variable contribution to risk. Variables on the left (red) are contribute to an increased risk of bleeding, while variables on the right (blue) contribute to a decreased risk of bleeding. Variables are organized such that those providing the strongest change to risk are at the center, with variables providing smaller changes to risk at the outside. (DOCX) [file pdig.0000906.s025.docx]

**S17 Fig.** SHAP explainer for Case Study B.
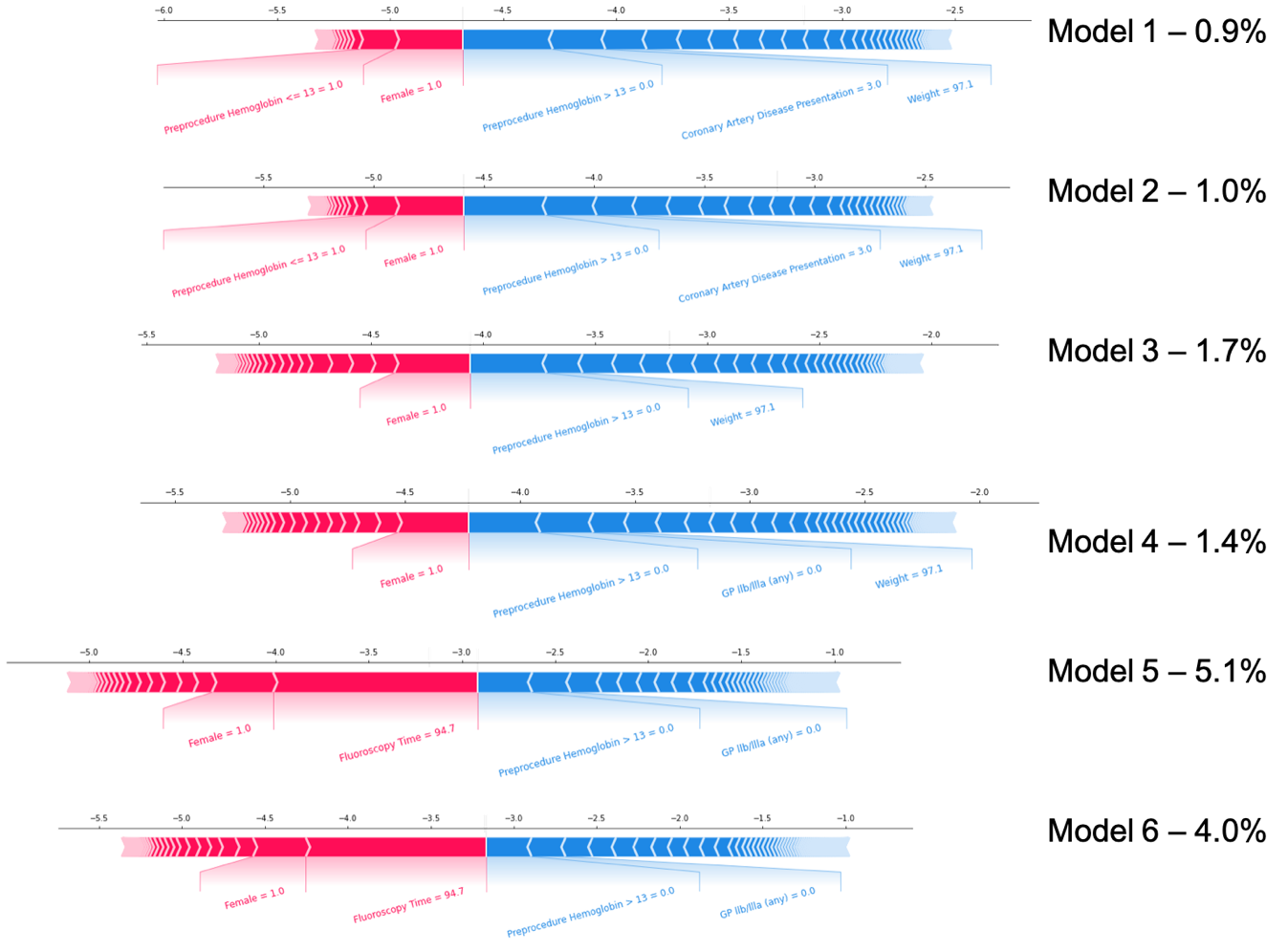

Supplement: S17 Fig — (DOCX) [file pdig.0000906.s026.docx]
