# Supplementary material for: Towards a dynamic model to estimate evolving risk of major bleeding after percutaneous coronary intervention
Source: PLOS Digit Health. 2025 Jun 25;4(6):e0000906. doi: 10.1371/journal.pdig.0000906 (PMC12193038; doi:10.1371/journal.pdig.0000906)
Supplement: S5 Text — (DOCX) [file pdig.0000906.s014.docx]

*Case Studies*

**S4 Table** shows the different values of **Table 1** when divided by those with and without bleeding cases. The variables used for the study are shown in **S1 Table**.

*Case Studies*

Because severe bleeding is a relatively rare event, risk changes for a minority of patients. However, some patients’ risk changes dramatically throughout the course of their care. Overall, the median difference of risk from the initial to final prediction is -0.41% (IQR -1.16%, +0.02%). However, the full range of risk changes was much larger, ranging from -44.4% to +83.2%. Two illustrative examples of more drastic changing risk is demonstrated in **S15 Fig** and described below.

Case Study A

A man in his 60s presented for emergent PCI for STEMI. In the initial model, his risk of bleeding was estimated to be 4.3%. This risk was driven predominantly by the emergent need for PCI, preprocedural hemoglobin, presence of STEMI, sex, and weight. The decision was made to use radial access, after which his bleeding risk was estimated to be 2.8%. There were no adverse findings during diagnostic coronary angiography, and risk decreased to 2.1%. He received prasugrel and unfractionated heparin, after which his risk fell to 1.5%. Following successful PCI, his risk further fell to 1.2%. His access site was closed using a suture-based closure device, and the final bleeding risk was 1.0%. This patient did not experience a bleed. Plots of individual SHAP values for this patient at each model stage are shown in **S16 Fig.**

Case Study B

A woman in her 60s presented for elective PCI for stable angina. In the initial model, her risk of bleeding was 0.9%. This risk was driven predominantly by her preprocedural hemoglobin, sex, stable nature of her coronary artery disease, and weight. Femoral access was used, after which her risk increased to 1.0%. Upon diagnostic coronary angiography, it was discovered that she had significant coronary stenosis present. The model at this stage estimated risk of bleeding to be 1.7%. She received unfractionated heparin and clopidogrel, after which her estimated risk was 1.4%. PCI was notable for high complexity (reflected by a long fluoroscopy time), after which her risk of bleeding increased to 5.1%. Her access site was sutured, and her final risk was 4.0%. This patient experienced a post-PCI bleed. Plots of individual SHAP values for this patient at each model stage are shown in **S17 Fig.**
