# Supplementary material for: Towards a dynamic model to estimate evolving risk of major bleeding after percutaneous coronary intervention
Source: PLOS Digit Health. 2025 Jun 25;4(6):e0000906. doi: 10.1371/journal.pdig.0000906 (PMC12193038; doi:10.1371/journal.pdig.0000906)

**S15 Fig.** Plot of case study risk scores across all model stages.

Case Study A began as high risk but was low risk in the final model. Case Study A did not ultimately bleed.

Case Study B began as low risk but was high risk in the final model. Case Study B ultimately experienced a bleed.
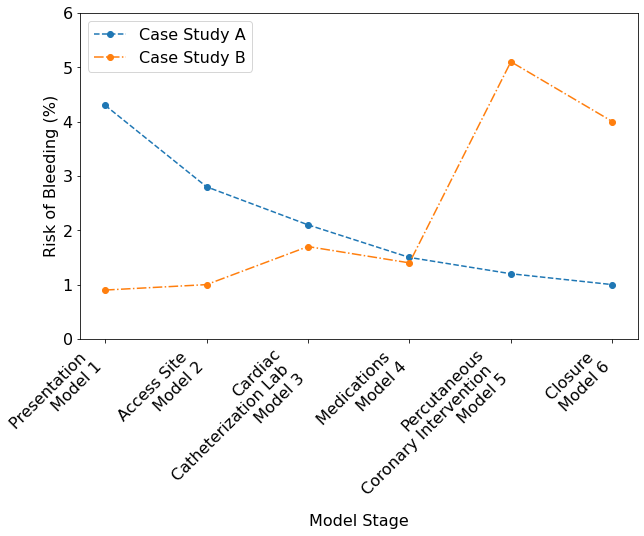

Supplement: S14 Fig — (DOCX) [file pdig.0000906.s023.docx]
