# Supplementary material for: Towards a dynamic model to estimate evolving risk of major bleeding after percutaneous coronary intervention
Source: PLOS Digit Health. 2025 Jun 25;4(6):e0000906. doi: 10.1371/journal.pdig.0000906 (PMC12193038; doi:10.1371/journal.pdig.0000906)

**S16 Fig** SHAP explainer for Case Study A. At each model stage, the prediction is created by summing each variable contribution to risk. Variables on the left (red) are contribute to an increased risk of bleeding, while variables on the right (blue) contribute to a decreased risk of bleeding. Variables are organized such that those providing the strongest change to risk are at the center, with variables providing smaller changes to risk at the outside.
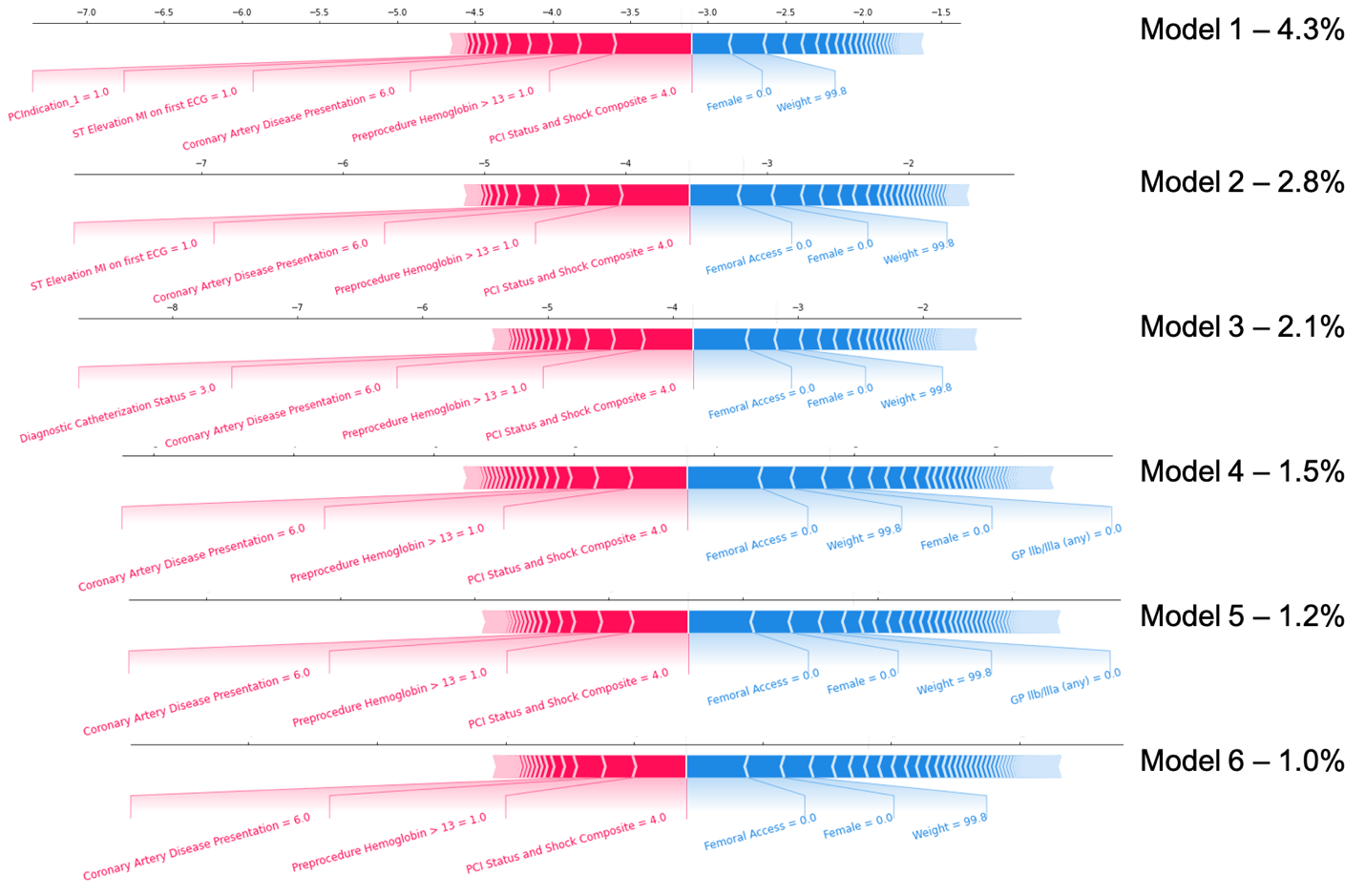

Supplement: S15 Fig — Case Study A began as high risk but was low risk in the final model. Case Study A did not ultimately bleed. Case Study B began as low risk but was high risk in the final model. Case Study B ultimately experienced a bleed. (DOCX) [file pdig.0000906.s024.docx]
