# Supplementary material for: Towards a dynamic model to estimate evolving risk of major bleeding after percutaneous coronary intervention
Source: PLOS Digit Health. 2025 Jun 25;4(6):e0000906. doi: 10.1371/journal.pdig.0000906 (PMC12193038; doi:10.1371/journal.pdig.0000906)
Supplement: Attachment — Submitted filename: Dynamic_Bleeding_Reviewer_Comments_auresp_2.docx [file pdig.0000906.s028.docx]

Dear editors and reviewers,

We would like to thank you for the time and effort into considering our paper. We were happy to receive the helpful comments that we have since addressed in revision. We believe the collective set of answers strengthens our work and better contextualizes our findings and clarifies issues surrounding variable selection. We provide a response to those comments in letter here below as well as in a tracked changes format of the manuscript re-submitted for your consideration.

Thank you,

Bobak Mortazavi

On behalf of all the authors

--

**Reviewer 1:**

The author has addressed many concerns; however, further consideration of multicollinearity is necessary. Specifically, the handling of the following variables may not be appropriate, leading to unstable results and making the SHAP analysis difficult to interpret. It would be advisable to reconsider these aspects.

**[R1]** Firstly, if AGEGT70 and AGELE70 have opposite meanings, retaining both variables will cause multicollinearity. The following binary variables may also raise similar concerns:
・OnsetTimeEst & OnsetTimeNA
・PREHGBGT13 & PREHGBLE13

**Response:** We thank the reviewer for raising this issue again and helping us dive deeper into the modeling, allowing us to further clarify what we are demonstrating and what we are showing as results. While we disagree somewhat with the collinearity being an issue because of the approach we are taking and the results we are trying to show, we realize the need to clarify all these matters in the manuscript. Here we describe the thought process and modeling a bit futher, including running the experiments you suggest, and then show all the places we have modified the manuscript and the supplementary material, all now improved and clearer in motivation and findings because of your comments!

While we understand that with Logistic Regression, multicollinearity is a problem, these cases are not a challenge to decision tree models. Whereas in regression analysis, multicollinearity is a problem and interaction terms would have to be created and explicitly tested, the modeling technique employed by XGBoost does not face such challenges. In particular, the broader array of variables allows XGBoost to discover the interactive terms that are predictive to each subset of the training region that it is going through in a divide and conquer technique. Further, our intention in this manuscript was to demonstrate that addition of variables and time to the existing methods would demonstrated further risk stratification. By removing features we introduce another variable that confounds interpreting the findings. These features with opposite meaning exist in the published model by Rao et al.

However, we understand the reviewers concerns and so we have conducted experiments where we have removed the colinear terms. In particular, we have removed all the derived dichotomous variables from their continuous and categorical counterparts, denoted with highlighting in the table provided in the Supplementary Material. What we find aligns with our intuition, the reduction of these variables does not significantly reduce performance, but given the broad set of data, categories and decision trees designed by XGBoost, there is a reduction in performance.

We copy some of the results here for you, then we will discuss the changes we have made to the manuscript.

While we see the same trends in increased performance with increased information over time, all results are notably worse. The reason for this is clearly the method by which XGBoost learns slowly, with a sequence of weak learners each with access to the full complement of their own features.

That said, we agree with the reviewer that if this concern remains this should be discussed in the manuscript. As such we have made changes to the main text and the supplementary material to address the performance with and without these multicollinear features.

In the introduction we have added the following:

*Accordingly, we sought to develop models that update estimates of patient risk of bleeding over time, enabling a dynamic estimate of risk that incorporates evolving clinical information. Taking the variables from the prior published risk models that used logistic regression and boosted decision trees from a wide compliment of available variables ^3,4^, we sought to demonstrate that risk stratification changes over time and notion of available data. In addition, we then sought to define a parsimonious model, with a number of colinear factors removed in order to compare findings.*

We have added this entirely new discussion in the results sections:

*Models were trained in a stratified, five-fold cross validation. Model performance metrics at each stage are provided in* ***Table 2****, and each stage is described further below. The model area under the receiver operating characteristic curves (AUROCs) increase from 0.812 to 0.845, and model area under the precision recall curves (AUPRCs) increase from 0.203 to 0.242, respectively. These results included decision tree models that use all available data for risk stratification.* ***Supplementary Table 2*** *lists all the variables used in our XGBoost-trained model. We maintained a number of extracted features persistent across prior models by Rao et al.^3^ and Mortazavi et al. ^4^, in order to demonstrate that all the available variables, when considered in a staged setting, allow further clarity towards the dynamic nature of Risk of Major Bleeding. We also sought to evaluate if the multicollinearity of these variables impacted the modeling, as a separate experiment, in order to clearly identify performance gains as a result of variables versus performance gains as a result of the dynamic staging of models. By removing all the created, binary indicator features, leaving only their continuous or categorical variables from which they were extracted, we actually saw a slight decrease in model performance, with AUROCs ranging from 0.808 to 0.843 and AUPRCs from 0.198 to 0.237. Because of the way the gradient boosted decision trees model each weak learner across the participants in the training dataset, it makes sense that the non-linear risk is better modeled with a wider array of features, some continuous, some categorical, and some extracted binary daughter variables. Further detail of this additional experiment is described in the* ***Supplementary Material.***

We have added the further explanation in the methods section:

*We recognize that the variables have multicollinearity, particularly the derived indicator features based upon the continuous feature counterparts. As this work sought to demonstrate changes in risk over time, we did not remove any variables used in the prior two studies, as we sought to only change the time and addition of new variables at each stage in evaluation. As a result, we acknowledge some co-linearity of variables as a potential limitation in performance and in interpretation. As a result, the* ***Supplementary Material*** *also discusses the same conducted analysis with a reduced feature set, where those particular indicator features have been removed, and we find that performance is comparable, though slightly stronger when including all possible predictors for the model to explore the decision space with.*

We have also added the following detailed section in the Supplementary Material, including the two figures above:

Multicollinearity Analysis

The primary purpose of this manuscript was to take the data available to the prior models and show that if you add data over time, the staged analysis shows changes in risk stratification. However, one might look at the data and notice both continuous variables (such as pre-procedure hemoglobin) and derived, indicator daughter variables (e.g. pre-procedure hemoglobin less than or equal to 13). An advantage of XGBoost is that the divide and conquer approach it takes towards building weak learners allows it to find non-linear relationships between the best predictive features given the particular subset of participant data each tree is being trained on. This has an advantage where a traditional logistic regression would not, being able to handle many similar, collinear variables such as these indicators or missing/not drawn features. However, to be sure that these are not adversely impacting modeling, we conducted an analysis where we removed the collinear features that may cause confusion from **Supplementary Table 2**, highlighted, and re-ran our analysis. **Supplementary Figures 10 and 11** show the AUROC and AUPRC calculations. As noted, these do similarly but slightly worse. This is likely because the continuous and categorical parent variables are stronger predictors over the entire population, but given the depths of the trees and the number of weak learners built to make the strong XGBoost model, it is likely that the interaction between the daughter variables and some of the other features are clearer predictors for some subsets of participants in the dataset. This drop in performance shows that clinical expertise injected into feature creation and usage can still support a number of these models. Similarly, **Supplemental Figures 12-17** show the SHAP explainer plots for each model stage. The primary predictors remain consistent. An interesting finding is the inclusion of Pre-procedure hemoglobin and its two daughter dichotomous indicator variables in the primary analysis, and just pre-procedure hemoglobin in this analysis. As can be seen in these SHAP plots, the risk that this value provides does not monotonically increase or decrease with the hemoglobin value. This is why in the primary analysis we see both Pre-Procedure Hemoglobin > 13 and Pre-Procedure Hemoglobin <= 13 having potentially increasing risk. Unfortunately, SHAP is not able to show which of these features interact with each within the depths of the learned trees or coverage over the trees that would help further illustrate interpretability. Ultimately, a parsimonious model would need to be built with available predictors in an interpretable fashion to drive clinical understanding, but given the registry data available here in a retrospective analysis, this work focused on further highlighting need to think about time-varying data when conducting risk stratification.

**[R2]** Notably, in Figure 2, both PREHGBGT13 and PREHGBLE13 appear. Given that these variables have opposite meanings, why do both higher feature values(1=yes?) show higher SHAP values? Generally, if variables have completely opposite meanings, their SHAP values would also be expected to be opposite.

**Response:** We thank the reviewer for this comment. This is a result of a slightly confusing matters. Because of the non-linearity presented by the decision trees, the opposite variables do not have to have opposite contributions, specifically for the trees that they’re selected. SHAP unfortunately does not highlight the coverage of the features and so there is definitely an interpretation shortcoming when it comes to explaining XGBoost models. Because of the above multicollinearity discussion, we sought to clarify some additional feature importance by building SHAP explainers for all the stages for the model where we removed such features, per your comments above, and we have added this to the Supplementary Material as well. Further, highlighting the importance of your comments, we have sought to clarify the text on this particular matter.

Here is the added text in the main body – in addition to the plots and text added to Supplement discussed in response to the question above:

*SHAP feature importance plots for the Presentation Model and the Closure Model are in* ***Figure 2*** *(with intermediate models shown in* ***Supplementary Figures 3-6,*** *and SHAP plots with the colinear variables removed in* ***Supplementary Figures 12-17****). In each plot, the variables are sorted in order of decreasing importance. The color of a variable relates to the value of that variable, while location along the x-axis represents how much that variable contributes to the risk of bleeding. Features most strongly driving predictions of high bleeding risk appear on the right with high SHAP values, while features most predictive of low bleeding risk appear at the left. The specific plots, per stage, are described further below. Note that the plots do not show the interactions across the depth of the tree but present a broader view of overall risk per feature.*

**[R3]** Similarly, for AdmtSource, which has three categories: "ED," "Transfer," and "Other," when decomposed into a one-hot encoding, not creating two variables will result in multicollinearity. The following categorical variables may also raise similar concerns:
・Dominance_1, Dominance_2 & Dominance_3

**Response:** Again, we thank the reviewer for this comment. We understand that traditionally, such variables only have two indicators, with the third being accounted for in the intercept. However, with so many different cases and different trees, we wished to have each variable specifically there to help determine if/when the feature specifically drives risk in any particular direction for any subset of the participants in the dataset. As such, we left those variables the same, though as we noted in response to the first comment above, when there was a categorical variable that had the sub-category indicator variables, we did conduct the analysis without those one-hot indicators.
