# Supplementary material for: Towards a dynamic model to estimate evolving risk of major bleeding after percutaneous coronary intervention
Source: PLOS Digit Health. 2025 Jun 25;4(6):e0000906. doi: 10.1371/journal.pdig.0000906 (PMC12193038; doi:10.1371/journal.pdig.0000906)
Supplement: Attachment — Submitted filename: Dynamic Bleeding Reviewer Comments.docx [file pdig.0000906.s027.docx]

Dear editors and reviewers,

We would like to thank you for the time and effort into considering our paper. We were happy to receive the helpful comments that we have since addressed in revision. We believe the collective set of answers strengthens our work and better contextualizes our findings and we provide a response to those comments in letter here below as well as in a tracked changes format of the manuscript re-submitted for your consideration.

Thank you,

Bobak Mortazavi

On behalf of all the authors

--

Editor Comments:

**[E1]** The 3 peer reviewers had somewhat different assessments. Collectively, they have identified a number of issues. Many of those involve analytical methods, or at least how those methods are described. The feature selection process should be described in more detail. It appears that some of the features are highly correlated, which may introduce unnecessary redundancy to the model, as pointed out by Reviewer 1.

**Response:** We thank the editor, and reviewer, for the comments. We agree that there is a lot of potential collinearity in the data selected. Our primary goal of this work was to take the existing work, and expand upon it rather than build from ground up. This meant taking the existing data, separate it in stages, and at most add variables. Therefore we demonstrate performance gains as a function of time and additional data. Therefore we did not remove any data. We provide further detail/justification below for the reviewer. Additionally, XGBoost does well with a variety of data available to it to be able to best model all the individual cases in the weak learners it trains.

If the editor and reviewer still believe the models should be re-run with some additional feature selection prior to the self-selection by the model we will happily run additional supplementary analysis but we feel the selection of key features aligns with some of the requests of Reviewer 3 to discuss real-world, real-time implementations in future work. To further discuss this please see responses

**R1-3, R1-5, and R3-5**

**[E2]** A data drift issue has also been raised. Although using older datasets does not diminish the merit of the study, the risk of data drift is a limitation worth mentioning.

**Response:** We completely agree data drift issue is potentially an issue. We split by time because we wanted to demonstrate that the model gains are found even with respect to potentially changing clinical workflows/time. Please see response **R3-5** for more discussion.

Again if the reviewer and editors wish for us to also cross validate with a random shuffle of subjects we will do that.

**[E3]** They also requested further explanation on how the model would be implemented in clinical practice.

**Response:** We have added key discussion points around this in response to the reviews. Please see response **R3-8,** below.

--

**Reviewer 1:**

Nathan Hurley et al. used a large database to build a dynamic model to assess the risk of major bleeding after PCI. In clinical practice, dynamic patient assessment is important, and the authors' approach is fascinating.

However, there are several, analytical and clinical concerns that need to be addressed as follows;

**R1 -1.** The first and most important issue is the definition of the outcome. Major bleeding is defined as occurring after the start of PCI. Since some of the predictors are obtained during PCI, these features no longer seem to be predictive. The outcome should be defined after the predictors are obtained.

**Response**: We thank the reviewer for this comment and dive in deeply into what we have discussed. We note that we already highlighted this as a key limitation to the latter models:

*“The second is the timing of the available variables. The registry abstracts some variables as pre- and intra- procedure, so some confounding may exist from reactions to bleeds during the procedure.”*

But further we will discuss why this is not a significant barrier to the findings of the paper. Indeed, major bleeding can be up to 72 hours after and while we would think to modify the outcome variable we wanted to stick with the traditional variable. We did not change this definition to adhere to the outcome currently used in practice by the NCDR models. In clinical traditional models have done this and we are meeting the same definitions/approaches, for example in considering approach and closure mechanism (e.g., femoral and closure). However, the innovative component of our work here is that the dynamic nature allows for continually updating risk as new predictors are obtained. If only predictors up to a certain point are available, that stage of the model is appropriate to use and our approach is the same. However, if new predictors are available and bleeding has not yet occurred, then our dynamic model takes them into account. Now, with respect to the results, we will highlight this point in the approach and results and discuss the limitations of the last stage model, specifically, but this is the only model potentially impacted by this and determining the change in association in risk factors. The changes are noted below for your record:

*“The staging of models is designed to be aligned with key clinical decision-making points. However, by the definition of major bleeding, it is possible that these are confounded with periprocedural bleeding rather than bleeding that can take place up until 72 hours after the procedure. As a result, the final two models can only be used as a decision making model if major bleeding has not yet occurred. “*

Additionally, in discussion:

*“That said, we still find same estimation improvements and risk changes through the addition of medication, which we believe supports our conclusions for needing a dynamic model.”*

**R1 -**2. Hemoglobin was a fairly high predictive variable in the results. First, what makes Hb over 13 a high risk in terms of pathology? What concerns me is the validity of the definition of major bleeding as a decrease in Hb 3 or higher. For example, is it possible that patients with Hb 13 or higher could be dehydrated? The decrease of Hb by fluid infusion is often seen in patients with higher Hb. Since transfusion and intervention/surgery at the bleeding site were also defined as bleeding in this study, it might be helpful to perform sensitivity analyses to confirm that similar results are obtained with the outcome definitions without hemoglobin.

**Response:** We appreciate the reviewer for these comments however, they are deemed out of scope for our work. We approached this work as an extension of

3.	Rao SV, McCoy LA, Spertus JA, et al. An updated bleeding model to predict the risk of post-procedure bleeding among patients undergoing percutaneous coronary intervention: a report using an expanded bleeding definition from the National Cardiovascular Data Registry CathPCI Registry. *JACC: Cardiovascular Interventions*. 2013;6(9):897-904.

While we do agree that the definition itself could be altered, we leave that to other clinical submissions, as we wanted here to simply state that the nature of the predefined predictive variables captured in the NCDR registry do provide this dynamic risk.

**R1-**3. It seems to me that the author includes as many variables as possible. For example, insurance does not seem relevant to the assessment of bleeding risk, but is it necessary? If the authors think that these variables should be included, the rationale should be noted.

I am also concerned about the several similar variables(e.g. Hb >13, HB <13, and continuous Hb.).

Furthermore, several variables seem to be the same variable(e.g. FEMALE and Sex, RENFAIL and CKD4).

Please provide reasons why these variables are included.

**Response:** We thank the reviewer for this comment, agree, and explain why we have kept some variables, removed some others (and re-run analyses), and trusted the model to eliminate some.

First, allow us to briefly explain the sequence of developments towards this work.

1) 3.	Rao SV, McCoy LA, Spertus JA, et al. An updated bleeding model to predict the risk of post-procedure bleeding among patients undergoing percutaneous coronary intervention: a report using an expanded bleeding definition from the National Cardiovascular Data Registry CathPCI Registry. *JACC: Cardiovascular Interventions*. 2013;6(9):897-904.

Was developed using these predefined, dichotomous splits.

Our prior work

2) 4.	Mortazavi BJ, Bucholz EM, Desai NR, et al. Comparison of machine learning methods with national cardiovascular data registry models for prediction of risk of bleeding after percutaneous coronary intervention. *JAMA network open*. 2019;2(7):e196835-e196835.

Sought to show that the models could perform better using the full predictive range of variables. However, there are still cases in which certain expert-guided features matter. As a result you see both the thresholded “Hgb > 13” and continuous Hgb as the top variables in Figure 2, for example.

This work looked only to evaluate the timing and change in risk associated with the variables to show that risk should be more continuously calculated. When we originally considered modifying it, we looked back and reviewer concerns that were raised in (4) that did not want too many factors changed so that we could point to what improved at each stage. As a result, in this work, we left the variables the same where anything with all CAPS was our recreation of variables from (3). More importantly, XGBoost as a model creates a series of sequential trees built to model a variety of patients that were misclassified by prior iterations of tree building. As a result, keeping these variables is important because subsequent further trees may use the dichotomous split rather than the continuous variable in corner cases that have proven otherwise difficult to predict from the general prior trees/variables.

However, we agree with the reviewer that some of these are either the same or similar. We have removed any variable we may have added that had collinearity. Sex for example and rerun the analysis. Luckily results do not change as the model correctly feature selects and just maintains Female as the selected feature. Similarly, we have removed CKD4 since it is indeed RENFAIL. Similarly no change. We have left the remaining colinear variables in, if they were included as part of the prior study. We have added the following feature selection in the methods with the following text added to address these comments when discussing variables of interest:

*“We recognize that the variables have collinearity. However, as this work sought to demonstrate changes in risk over time, we did not remove any variables used in the prior two studies, as we sought to only change the time and addition of new variables at each stage in evaluation. As a result, we acknowledge some co-linearity of variables but removing these would only further enhance our findings.”*

As for insurance variables and others that may not be as predictive, we included these as potential surrogates for socio-economic status variables that often act as latent variables for other risks/conditions. If the reviewer ultimately still disagrees and wishes to see further models either in the paper or supplementary analysis we are happy to run those iterations as well including the inclusion/removal of some variables but we note now that we could have too many ablations across reviewer concerns for each of the specific variables and do believe that the next work, in which an ehr-native model is built, will consider variables more closely as a result of what is extractable from the

4. In addition, SHAP is not an analysis that can completely ignore multicollinearity, so it would be better to exclude unnecessary variables.

**Response:** While SHAP cannot ignore multicollinearity, the intention is not to have a single parsimonious working model. The registry data, as we note in our limitations, has too many restrictions to work in real-time as a dynamic model as we are building towards. What SHAP does show, even with this multicollinearity limitation, is that the top features are changing in each stage, illustrating our findings that there are significant changes in risk factors and model performance across stages.

**R1-5**. The risk of bleeding is quite different whether the access site is femoral or radial. Is it possible to show the difference in model performance in these subgroups?

**Response:** We agree with the reviewer that there should be differences and while we wanted to show overall associations, yes we can show differences in each subgroup. See the results below.

**Supplementary Table 4.** Comparison of model performances for bleeding prediction by subgroup on access site.

| **Model** | **Overall AUROC** | **Femoral AUROC** | **Radial AUROC** |
| --- | --- | --- | --- |
| **Presentation** | 0.812 (0.812-0.812) | 0.804(0.804,0.804) | 0.822(0.822,0.822) |
| **+ Access Site** | 0.817 (0.817-0.817) | 0.804(0.803,0.804) | 0.825(0.825,0.825) |
| **+ Cath Lab** | 0.825 (0.825-0.825) | 0.812(0.812,0.812) | 0.834(0.834,0.835) |
| **+ Medication Decision** | 0.832 (0.832-0.832) | 0.820(0.820,0.820) | 0.840(0.839,0.840) |
| **+ PCI Variables** | 0.844 (0.844-0.845) | 0.834(0.834,0.834) | 0.850(0.850,0.851) |
| **+ Closure Decision** | 0.845 (0.845-0.845) | 0.835(0.835,0.835) | 0.851(0.850,0.851) |

What we find here is, as expected, the prediction in Radial is easier (less likely to bleed). However the incremental gains found in the model are consistent across subgroups which is promising. We have also added the following text to our methods to point to these results in the methods section:

“*Knowing that radial and femoral access choice lead to different findings, we also conducted a subgroup analysis in which we evaluated model performance on each subgroup, discussed further in the* ***Supplementary Material.”***

And the following text in the supplemental material:

“*Knowing that radial and femoral access choice lead to different findings, we also conducted a subgroup analysis in which we evaluated model performance on each subgroup. As noted in* ***Supplementary Table 4****, we find that radial access expectedly performs better, likely because bleeds are less frequent and those that happen are clearer based upon key risk factors. However, what is of note here is that both femoral and radial see increased AUROC, with increased risk factor association with outcome over the stages and therefore the findings of the main models are consistent across the key subgroups here.”*

Reviewer 2:

Competent study examining the risk of major bleeding for patients undergoing percutaneous coronary intervention, showing the changes in patient risk estimation throughout the course of treatment. They demonstrate the need for more dynamic evaluation of risk estimates, providing potential changes in treatment decision making throughout admissions, accounting for prior treatment decisions made.

**Response:** We thank the reviewer for these comments, we are glad the reviewer found the changes in risk estimation insightful and demonstrative.

Reviewer 3:

**R3-1.** The study and manuscript have several areas that could be improved to enhance their overall robustness, applicability, and impact. One significant limitation is that validation was conducted using a temporal split within the dataset, without the inclusion of an external dataset.

To improve the generalizability of the model across different patient populations and healthcare settings, incorporating an external validation cohort is recommended. This could involve using data from a different registry or conducting a prospective study to assess the model’s performance in varied contexts.

**Response:** We completely agree with the reviewer that model generalizability needs to always be established for these kind of works, and while we acknowledge the ideal scenario would involve an external validation set, we do not believe that this particular dataset has such a suitable external validation. For one, This study included all index PCIs in the National Cardiovascular Data Registry (NCDR) CathPCI registry version 4.4 from July 2009 through April 2015, which contains patient data from over 1500 sites across the United States. This nationwide cohort consists of significant sites across a variety of hospital types and locations within the United States. External validation would be data from other countries, which do not have similar registry format for ease of translation. Varied contexts from this point forward would require a deeper dive into the hospital types found within the NCDR registry which, while important, is a different problem all together. Therefore, we find that the breadth and depth of the registry data is sufficient for the general findings discussed in this paper, in which a real EHR-based implementation of a real time model would have to show demonstrable proof across different specific hospital EHR implementations which we agree is important but also again it’s own work.

As for the temporal split vs. a cross-validation. We do not find this to be a significant limitation. In fact, we had originally done this for a different work using the registries and the clinical reviewers came back telling us that temporal splitting was preferred because generalizability has to also account for changes in workflows / procedures over time, and that a real generalizable model would identify key risk factors independent of certain changes. If the reviewer wishes, we are happy to put the standard cross-validation back into the results but we viewed the comment more as a concern on any form of validation within the NCDR and so believe our comments have addressed this. Further, we point to response to your question, R3-5 for more information on why the temporal split is beneficial.

**R3-2.** Another area for improvement is the integration of real-time data. The study acknowledges the challenge of acquiring real-time data and relies on manually abstracted data from the NCDR.

Future research could explore the integration of real-time data streams from electronic health records (EHRs) to continuously update the model's predictions. This would require developing robust methods for real-time data processing and ensuring the quality of the data being fed into the model.

**Response:** We agree completely that future work could AND SHOULD explore this integration. These models as they exist could be incorporated into an EMR system, allowing these predictions to be generated as these covariates are known. Ultimately, this work stops short of this implementation, but that would be the next logical step in this work. From our limitations section with added discussion based upon the reviewer’s comment:

*“The implementation of such a system at scale within an electronic health record environment, where data may be available within near real-time capacity, requires additional investigation of natural language processing techniques, models that handle advanced time-series data, and appropriate evaluation of user interface design for reducing clinician burden when interacting with such a model. This, additionally, would require an evaluation of the quality of the features being fed into the model to ensure those risk factors identified in this work are easily available and readily of high quality without the rigorous review the registry data undergoes.”*

**R3-3.**The study also discusses the need for reducing clinician burden when interacting with the model but does not explore this aspect in depth. Collaborating with clinicians to develop user-friendly interfaces and decision-support tools that seamlessly integrate into existing clinical workflows would be critical. Conducting user experience (UX) studies could ensure that the model's outputs are actionable and easily interpretable by healthcare providers.

**Response:** Similarly to R3-2, we agree that this would be a valuable future direction for this work. As written, this work focuses on establishing that this type of dynamic model would be possible, and future UX can be a future aim.

**R3-4.**While SHAP (SHapley Additive exPlanations) plots are used to interpret the model, the manuscript could provide more detailed explanations of how these insights could be applied in practice. Expanding on how clinicians can use SHAP plots to understand key predictors of bleeding risk and adjust treatment strategies accordingly would make the model more practical. Additionally, providing case studies or examples where the model influenced clinical decisions could help bridge the gap between machine learning outputs and clinical action.

**Response:** We agree that case studies can be useful in understanding this type of prediction. While this study was performed on retrospective data, future applications of this work in real-time would allow for clinicians to adapt to changing risk as patient situations change. We did add additional discussion on the variables as found, and how they change. We cannot say how the model influenced clinical decisions because this is a retrospective review of data. We will definitely do that in our future prospective work.

We have the following in the supplementary material in order to address the potential case studies:

*Case Studies*

***Supplementary Table 1*** *shows the different values of* ***Table 1*** *when divided by those with and without bleeding cases. The variables used for the study are shown in* ***Supplementary Table 2****.*

*Case Studies*

*Because severe bleeding is a relatively rare event, risk changes for a minority of patients. However, some patients’ risk changes dramatically throughout the course of their care. Overall, the median difference of risk from the initial to final prediction is -0.41% (IQR -1.16%, +0.02%). However, the full range of risk changes was much larger, ranging from -44.4% to +83.2%. Two illustrative examples of more drastic changing risk is demonstrated in* ***Supplementary Figure 7*** *and described below.*

*Case Study A*

*A man in his 60s presented for emergent PCI for STEMI. In the initial model, his risk of bleeding was estimated to be 4.3%. This risk was driven predominantly by the emergent need for PCI, preprocedural hemoglobin, presence of STEMI, sex, and weight. The decision was made to use radial access, after which his bleeding risk was estimated to be 2.8%. There were no adverse findings during diagnostic coronary angiography, and risk decreased to 2.1%. He received prasugrel and unfractionated heparin, after which his risk fell to 1.5%. Following successful PCI, his risk further fell to 1.2%. His access site was closed using a suture-based closure device, and the final bleeding risk was 1.0%. This patient did not experience a bleed. Plots of individual SHAP values for this patient at each model stage are shown in* ***Supplementary Figure 8.***

*Case Study B*

*A woman in her 60s presented for elective PCI for stable angina. In the initial model, her risk of bleeding was 0.9%. This risk was driven predominantly by her preprocedural hemoglobin, sex, stable nature of her coronary artery disease, and weight. Femoral access was used, after which her risk increased to 1.0%. Upon diagnostic coronary angiography, it was discovered that she had significant coronary stenosis present. The model at this stage estimated risk of bleeding to be 1.7%. She received unfractionated heparin and clopidogrel, after which her estimated risk was 1.4%. PCI was notable for high complexity (reflected by a long fluoroscopy time), after which her risk of bleeding increased to 5.1%. Her access site was sutured, and her final risk was 4.0%. This patient experienced a post-PCI bleed. Plots of individual SHAP values for this patient at each model stage are shown in* ***Supplementary Figure 9.***

**Supplementary Figure 7.** Plot of case study risk scores across all model stages.

Case Study A began as high risk but was low risk in the final model. Case Study A did not ultimately bleed.

Case Study B began as low risk but was high risk in the final model. Case Study B ultimately experienced a bleed.

**Supplementary Figure 8** SHAP explainer for Case Study A. At each model stage, the prediction is created by summing each variable contribution to risk. Variables on the left (red) are contribute to an increased risk of bleeding, while variables on the right (blue) contribute to a decreased risk of bleeding. Variables are organized such that those providing the strongest change to risk are at the center, with variables providing smaller changes to risk at the outside.

**Supplementary Figure 9.** SHAP explainer for Case Study B.

**R3-5.** Another consideration is addressing model drift. The model was trained and validated on data up to 2015 and we are in 2024, almost decade later, clinical practices and patient populations evolve over time, potentially leading to model drift. The manuscript could discuss strategies to monitor and update the model regularly to maintain its accuracy over time. This might involve periodic retraining with new data, continuous learning approaches, or deploying a feedback loop where the model’s predictions are compared with actual outcomes to recalibrate the model.

**Response:** We completely agree with this and up to date data would always be worthwhile. We did the temporal split in validation to try to show some things that evolve in clinical practice do not impact rates/findings in this work. We have added to our limitations some discussion of the time:

*“The NCDR relies upon manual chart abstraction for many variables, such as past medical history variables and as a result, data we have at our disposal is older, and at risk for data drift issues with respect to changes in clinical practice. Our best efforts to adjust for this was the temporal split. The implementation of such a system at scale within an electronic health record environment, where data may be available within near real-time capacity, requires additional investigation of natural language processing techniques, models that handle advanced time-series data, and appropriate evaluation of user interface design for reducing clinician burden when interacting with such a model.*  *This, additionally, would require an evaluation of the quality of the features being fed into the model to ensure those risk factors identified in this work are easily available and readily of high quality without the rigorous review the registry data undergoes.”*

**R3-6.** The study does not extensively discuss potential ethical concerns or biases that may arise from the use of machine learning in clinical decision-making. Including a section that addresses the ethical implications of dynamic risk models, such as potential biases (e.g., racial, gender, socioeconomic) and the need for transparent model governance, could enhance the manuscript. Strategies for bias detection and mitigation would help ensure that the model is equitable and fair across different patient subgroups.

**Response:** Indeed, bias concerns are great with ML models in health. For example:

Obermeyer Z, Powers B, Vogeli C, Mullainathan S. Dissecting racial bias in an algorithm used to manage the health of populations. Science. 2019 Oct 25;366(6464):447-53.

We use the national registry to be as generalizable across patient populations by containing as much information as possible. We included race, ethnicity, and insurance information to also account for potential socio-economic confounding variables. Perhaps beneficially, we do not see those variables come out as predictive.

**R3-7.** The primary outcome considered in the study is any in-hospital bleeding event within 72 hours after PCI. However, the manuscript could be improved by including long-term outcomes such as mortality, re-hospitalization, or recurrent cardiovascular events. This would provide a more comprehensive and practical view of the model’s impact on patient care and could involve exploring how the dynamic risk model correlates with these long-term outcomes.

**Response:** Unfortunately, we cannot have rehospitalization or mortality because this information is not available/linked to the registry data. We agree this would be extremely beneficial and would love to have such a dataset, perhaps within a single hospital system where patients are not lost to follow up.

**R3-8.** The discussion in the manuscript touches on the potential utility of the model but could delve deeper into its practical application. Expanding on how the dynamic model could be implemented in different clinical settings, such as tertiary care centers versus community hospitals, and its potential impact on clinical guidelines, would make the research more compelling. Additionally, discussing any cost-benefit analysis or resource implications of implementing such a model could be valuable.

**Response:** We agree with the reviewer and have added a paragraph in the discussion about how future models would be used prospectively based upon these findings. We paste that paragraph here for your ease of review:

*“This work enables future prospective modeling in electronic health record-native implementation. While we segmented the data on key decision points within the registry data, we demonstrate the potential for implementing a clinical decision making tool within the medical record for real-time use, where each available data point can be added for key stages within each hospital system’s clinical workflow. This would potentially benefit different clinical settings that have varied anti-coagulation efforts, ensure that the risk/treatment paradox with anti-coagulation is addressed, where those at highest risk are not being treated ^12^. This would provide the potential for biggest impact, identifying cases in real time that should or should not have bleeding avoidance therapies to help make continuous decisions in the best interest of patient outcomes.”*

12.	Rao SC, Chhatriwalla AK, Kennedy KF, et al. Pre-procedural estimate of individualized bleeding risk impacts physicians' utilization of bivalirudin during percutaneous coronary intervention. *Journal of the American College of Cardiology*. 2013;61(18):1847-1852.

**R3-9.** Finally, while the manuscript outlines future research opportunities, it could be more specific. The authors should consider provide concrete examples of how future studies could build on this work—such as by investigating specific subgroups, incorporating additional data types (e.g., genetic, imaging), or exploring alternative machine learning approaches—would give researchers a clearer roadmap for advancing this field.

**Response:** We agree please see response to R3-8 above.
